# Supplementary material for: Bacterial rhomboid proteases mediate quality control of orphan membrane proteins
Source: EMBO J. 2020 Apr 27;39(10):e102922. doi: 10.15252/embj.2019102922 (PMC7232013; doi:10.15252/embj.2019102922)
Supplement: Supplementary file 1 — Appendix [file EMBJ-39-e102922-s001.docx]

**Appendix**

**Table of Contents**

[Appendix Fig. S1. Identification of potential GlpG and Rhom7 substrates. 2-6](#_Toc33714575)

[Appendix Fig. S2. GlpG specifically targets orphan HybA. 7](#_Toc33714577)

[Appendix Fig. S3. HybA is cleaved by GlpG during the H_2_ consumption assay. 8](#_Toc33714578)


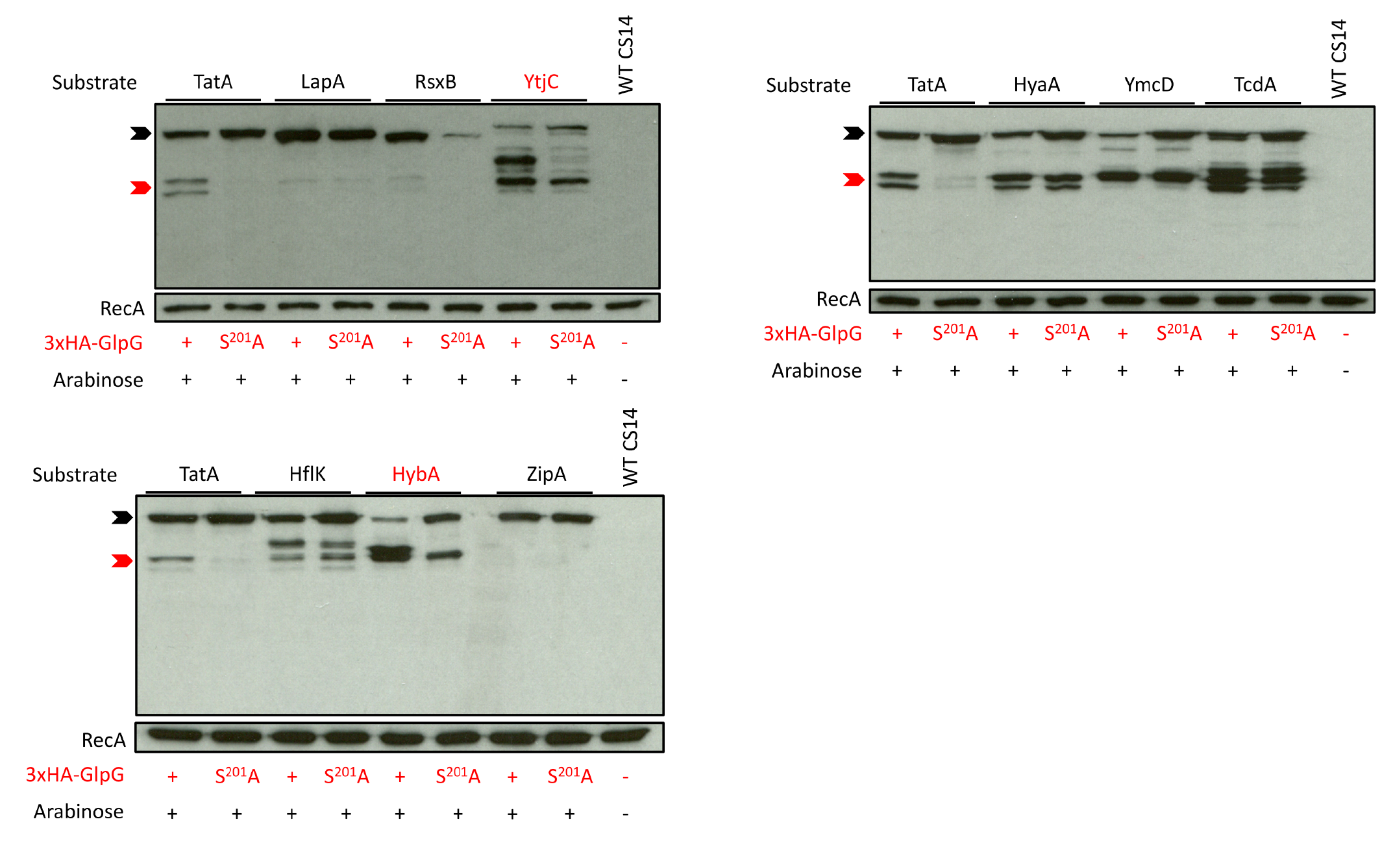

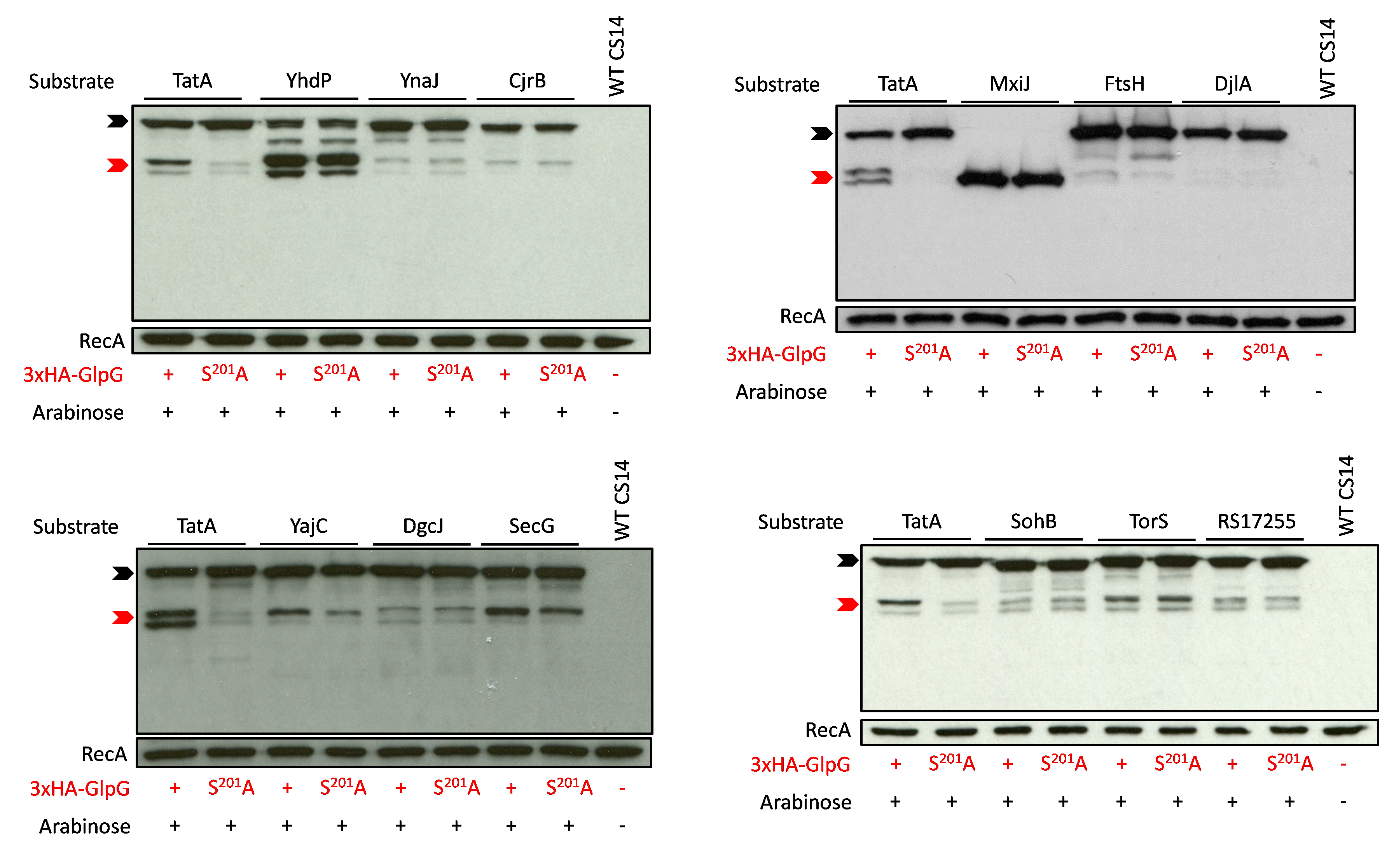

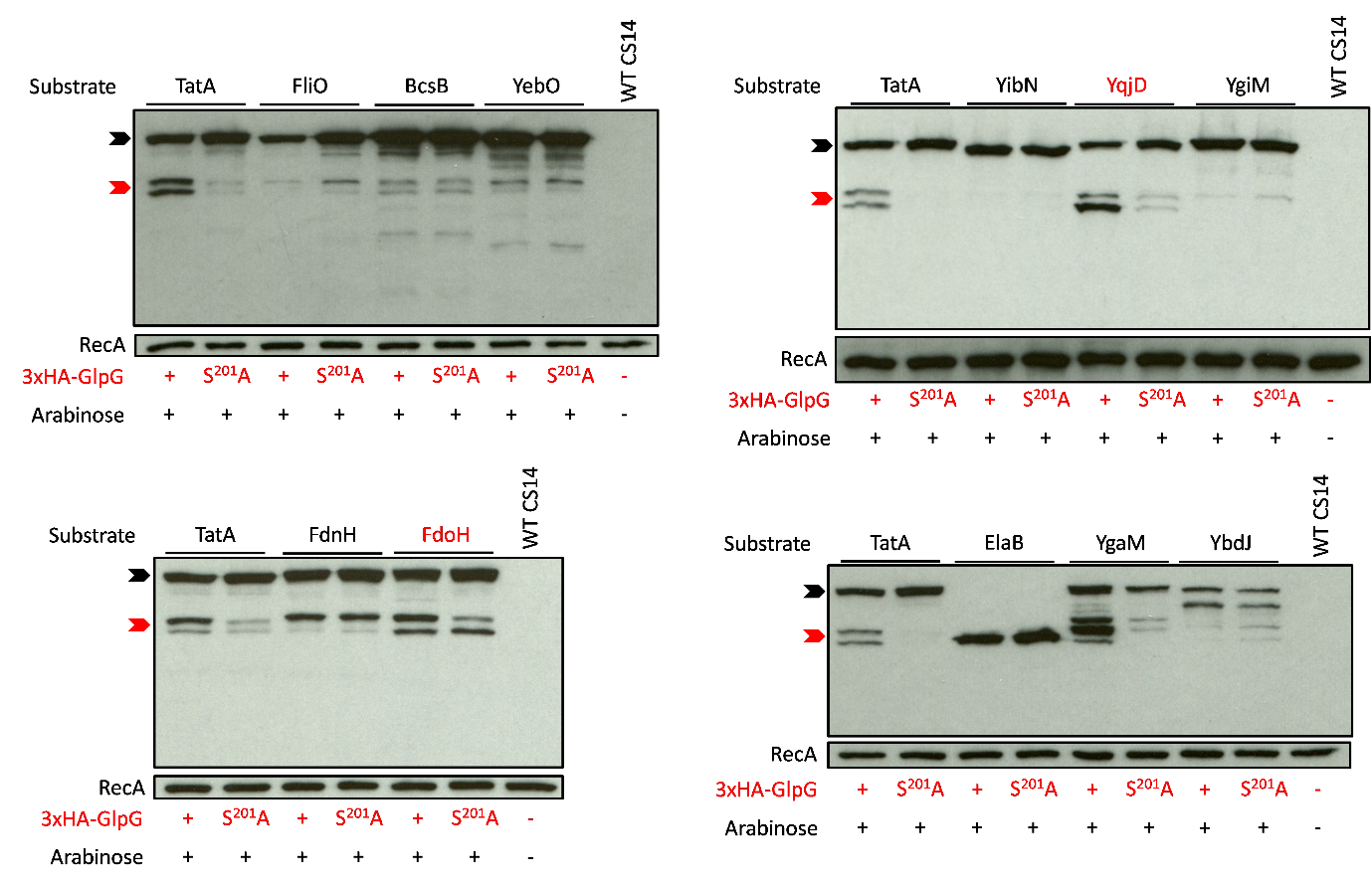

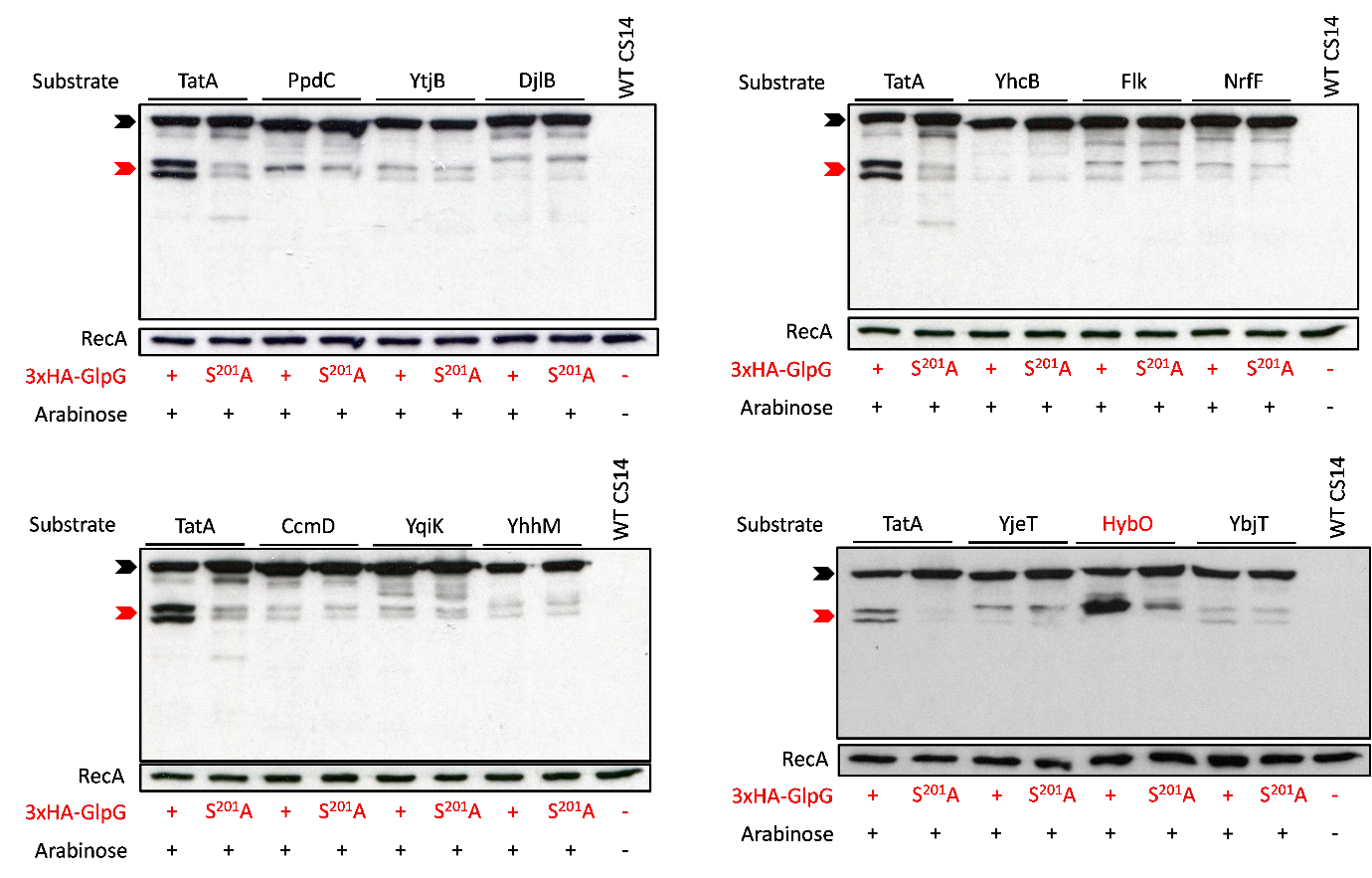

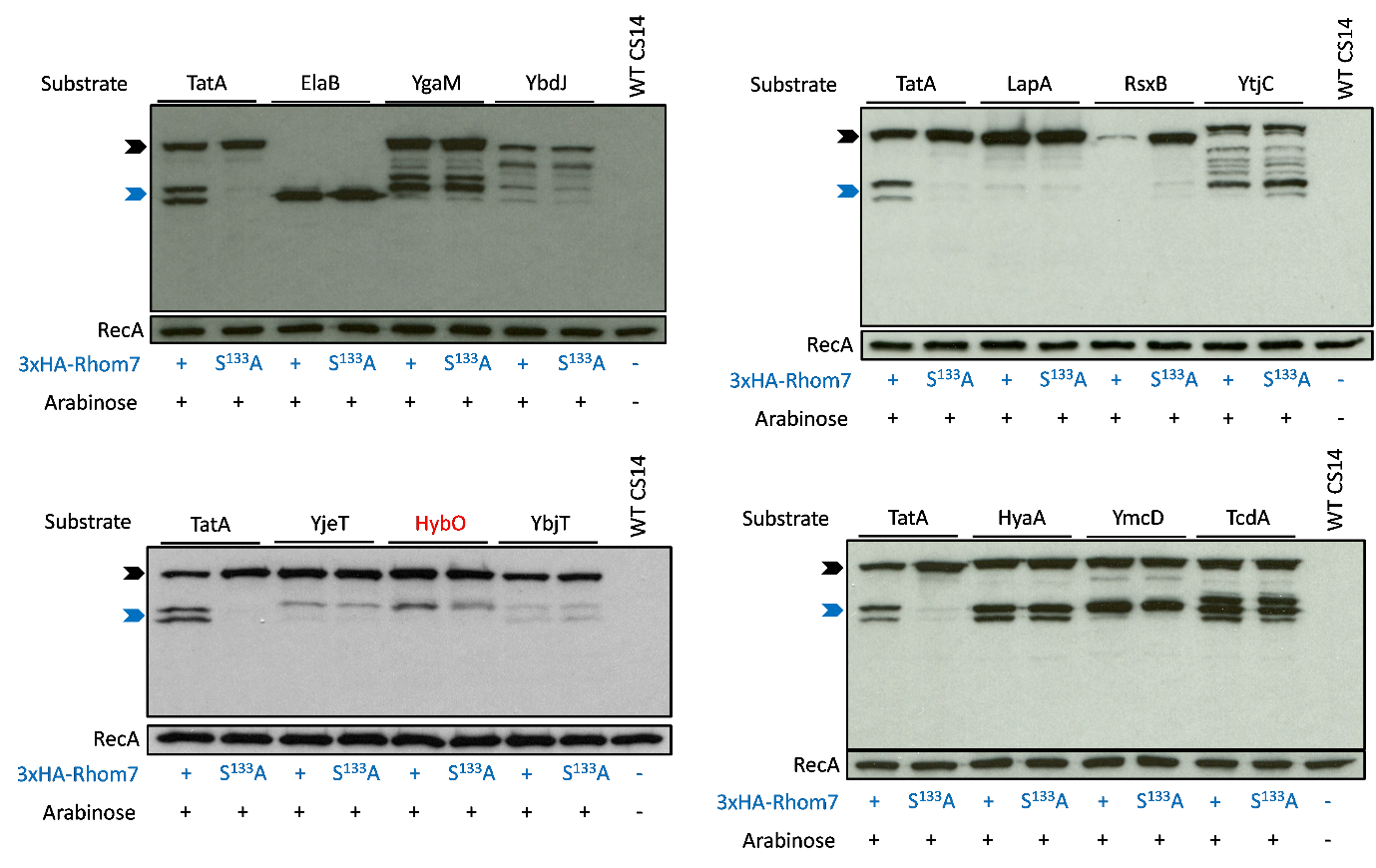

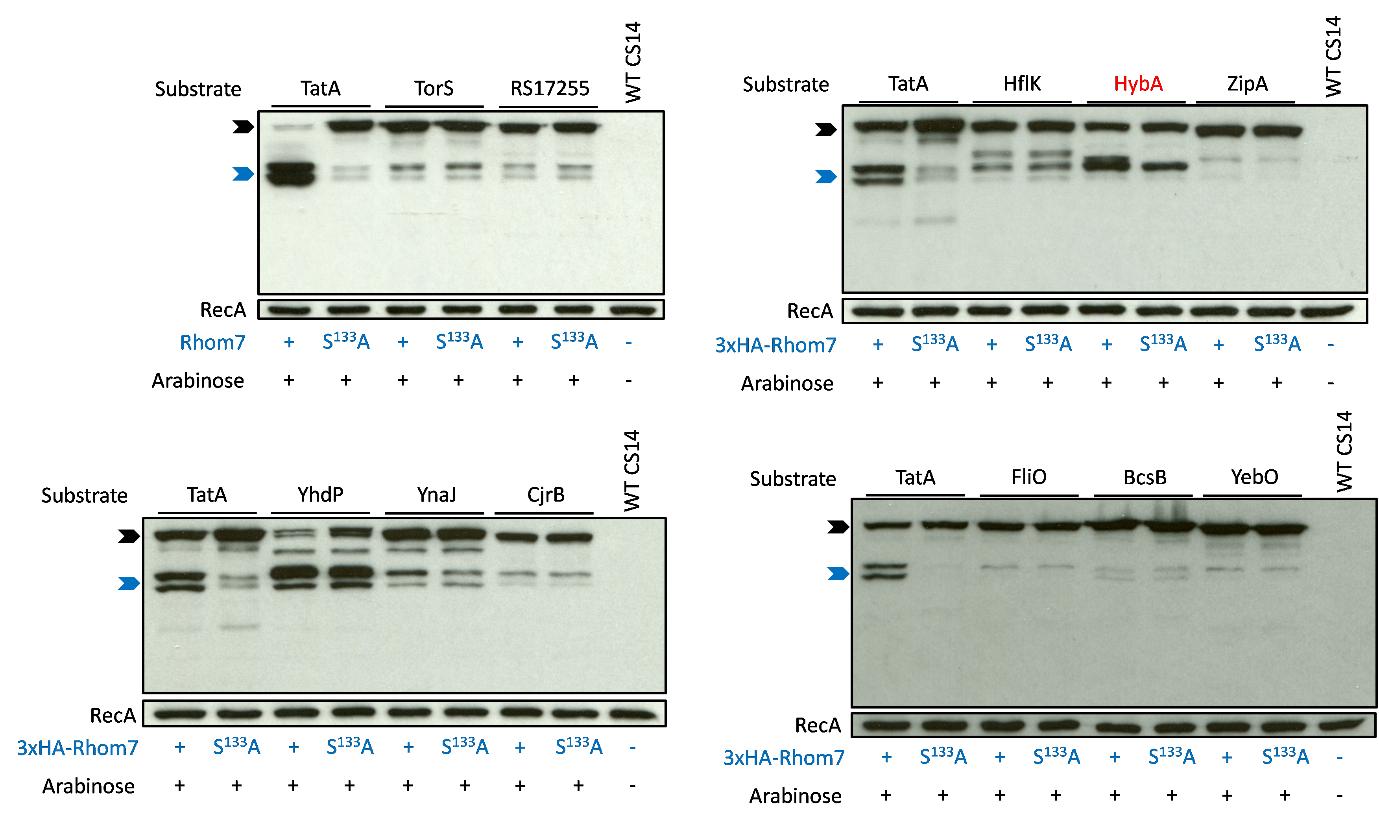

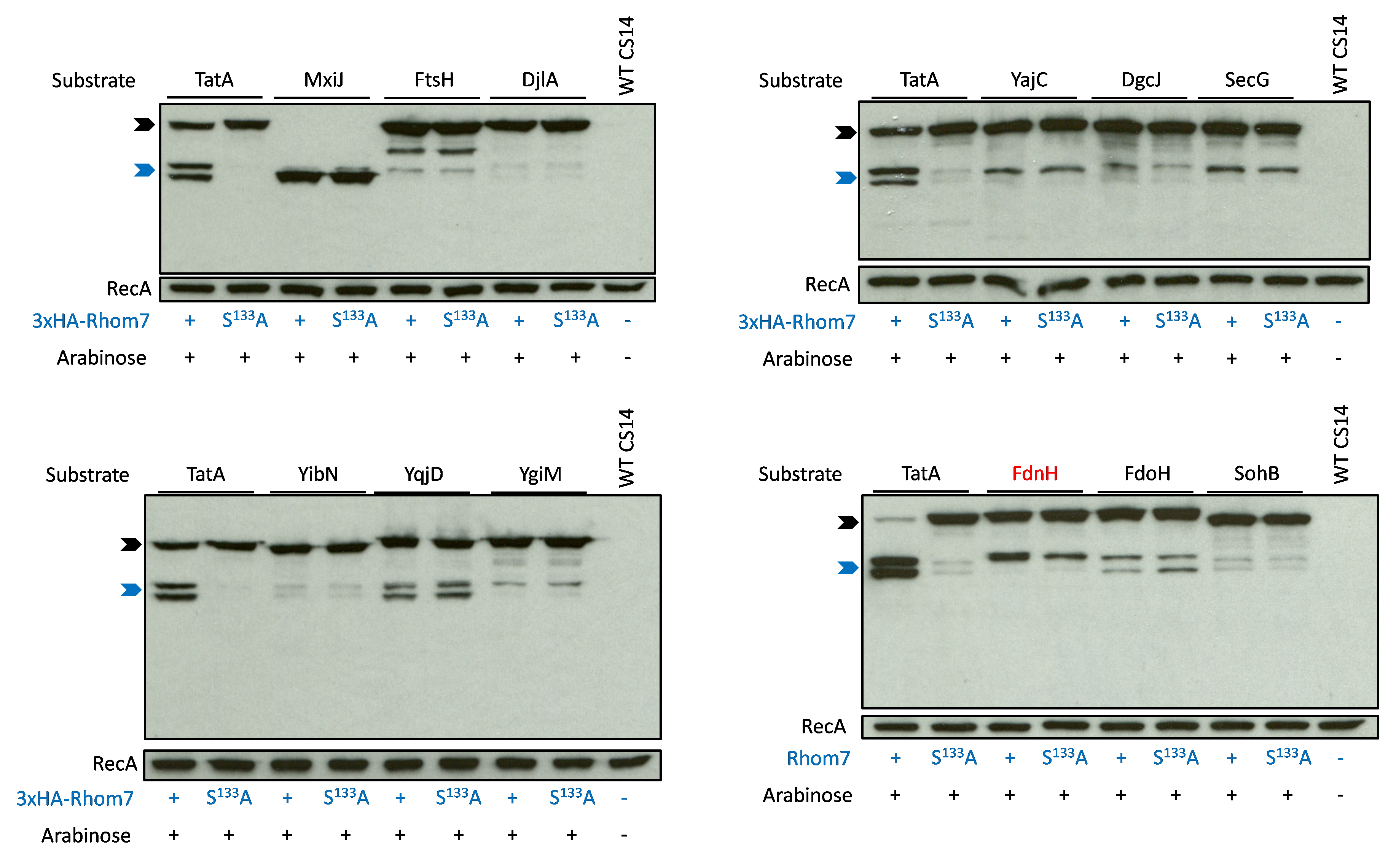


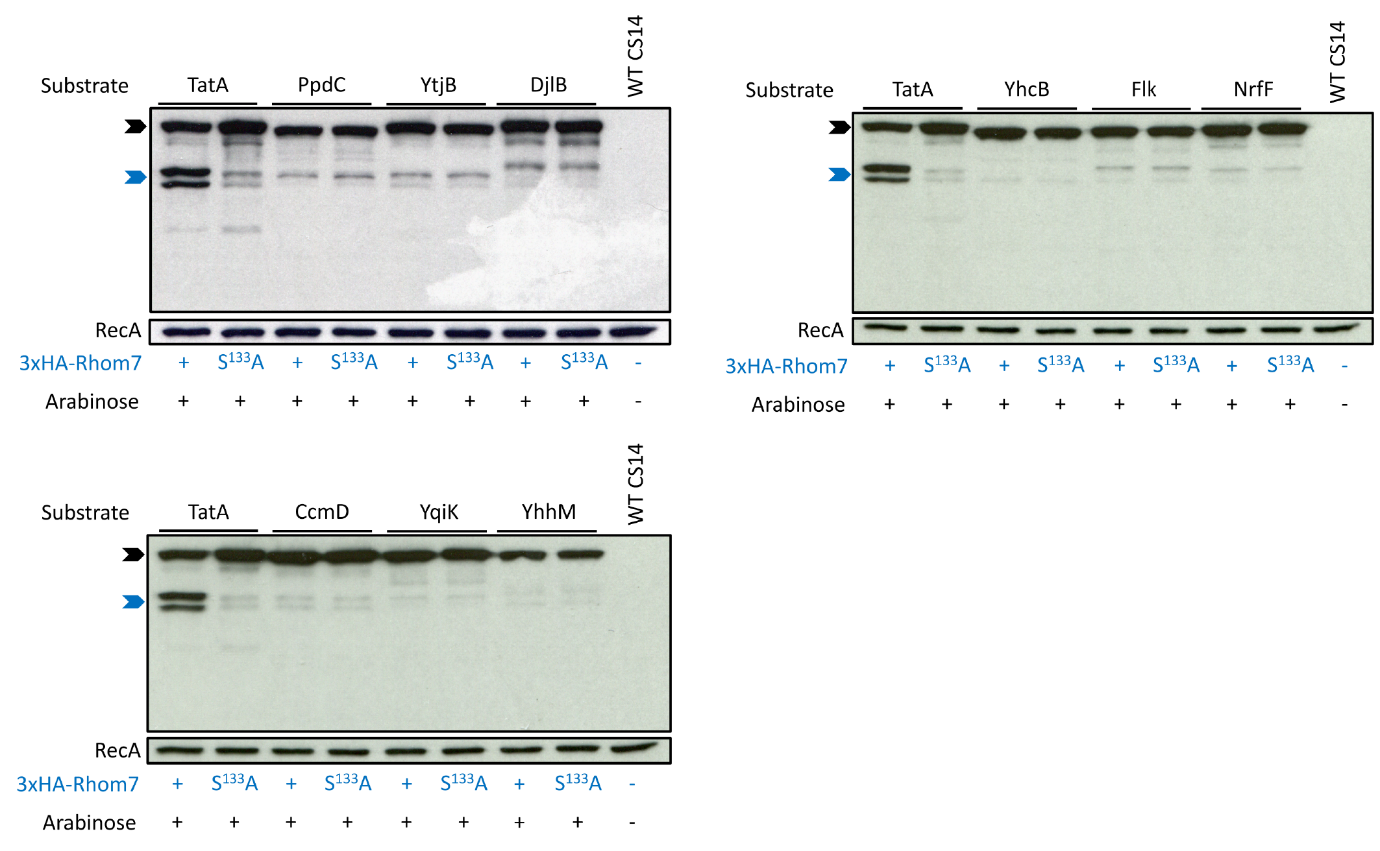


### ****Appendix Fig. S1****. Identification of potential GlpG and Rhom7 substrates.

### The screen was performed with *S. sonnei* Δ*glpG*Δ*rhom7* containing pKS508 encoding artificial substrates possessing TMDs from the 44 candidate substrates, and pBAD33 encoding triple HA-tagged (3xHA) wild-type (+) or inactive forms of GlpG (S^201^A) or Rhom7 (S^133^A). pBAD33-encoded Rhom7 (Rhom7) was not tagged in some experiments. Cleavage was assessed by western blot analysis using anti-FLAG antibodies. Hits (in red) are candidates for which there was an increase in the cleavage product (approximately 35 kDa) and a decrease in the amount of uncleaved substrate in the presence of the active enzyme. The TMD of *P. stuartii* TatA was used as a positive control. Candidate substrates that are uncleaved, cleaved by GlpG, or cleaved by Rhom7 are marked by black, red or blue arrows, respectively. RecA was used as a loading control.

**
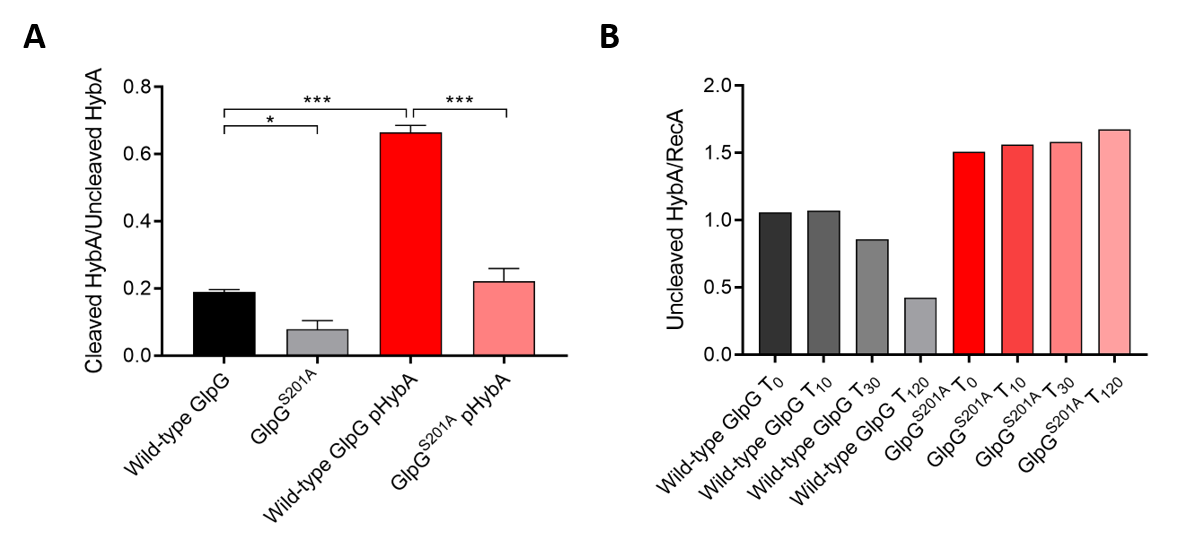
**

### ****Appendix Fig. S2****. GlpG specifically targets orphan HybA.

**A** Quantification of the ratio of cleaved HybA to uncleaved HybA in the presence or absence of over-expressed HybA (**Fig 4B**).

**B** Amount of uncleaved HybA (relative to RecA) in the presence wild-type or inactive GlpG (GlpG^S201A^) at times after blocking protein translation by the addition of chloramphenicol at T_0_ in the absence of HybB (**Fig 4D**).

Data information: P-values were calculated by one-way ANOVA with Tukey’s test (A). Bars represent the mean ± S.D. n = 2 (A); n = 1 (B). *, p<0.05; ***, p<0.001

###
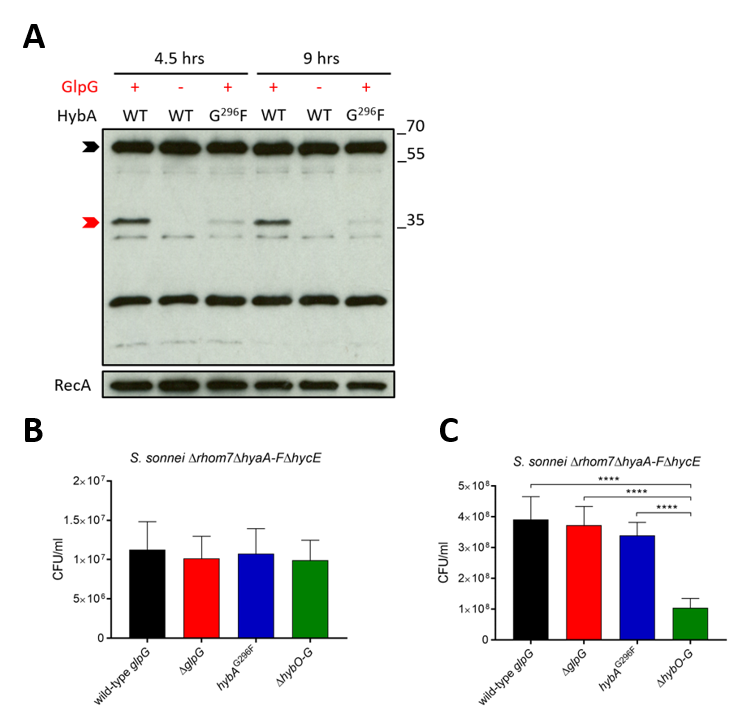


### ****Appendix Fig. S3****. HybA is cleaved by GlpG during the H_2_ consumption assay.

**A** Western blot analysis of cleavage of wild-type (WT) HybA or HybA bearing a G^296^F mutation (G^296^F) in *S. sonnei* in the presence (+) or absence (-) of chromosomally-encoded GlpG. Extracts were prepared from bacteria grown for experiments shown in **Fig 4G** (at T_4.5_ and T_9_). HybA that is uncleaved or cleaved by GlpG is marked by black and red arrows, respectively.

**B** The number of bacteria in the input at T_0_ in **Fig 4G**.

**C** The number of bacteria in the output at T_9_ in **Fig 4G.**

Data information: P-values were calculated by one-way ANOVA using Tukey’s test (B, C). Bars represent the mean ± S.D. n = 3 (B); n = 3 (C). ****, p<0.0001
