# Supplementary material for: Bacterial rhomboid proteases mediate quality control of orphan membrane proteins
Source: EMBO J. 2020 Apr 27;39(10):e102922. doi: 10.15252/embj.2019102922 (PMC7232013; doi:10.15252/embj.2019102922)
Supplement: Supplementary file 2 — Expanded View Figures PDF [file EMBJ-39-e102922-s002.pdf]

# Expanded View Figures

```
GlpG_Ec      MLMITSFANPRVAQAFVDYMATQGVILTIQQHNQSDVWLADESQAERVRAELARFLENPA  60
GlpG_Ss      MLMITSFANPRVAQAFVDYMATQGVILTIQQHNQSDVWLADESQAERVRAELARFLENPA  60
             ************************************************************

GlpG_Ec      DPRYLAASWQAGHTGSGLHYRRYPFFAALRERAGPVTWVMMIACVVVFIAMQILGDQEVM  120
GlpG_Ss      DPRYLAASWQAGHTGSGLHYRRYPFFAALRERAGPVTWVMMIACVVVFIAMQILGDQEVM  120
             ************************************************************

GlpG_Ec      LWLAWPFDPTLKFEFWRYFTHALMHFSLMHILFNLLWWWYLGGAVEKRLGSGKLIVITLI  180
GlpG_Ss      LWLAWPFDPALKFEFWRYFTHALMHFSLMHILFNLLWWWYLGGAVEKRLGSGKLIVITLI  180
             *********:**************************************************

GlpG_Ec      SALLSGYVQQKFSGPWFGGLSGVVYALMGYVWLRGERDPQSGIYLQRGLIIFALIWIVAG  240
GlpG_Ss      SALLSGYVQQKFSGPWFGGLSGVVYALMGYVWLRGERDPQSGIYLQRGLIIFALIWIVAG  240
             ************************************************************

GlpG_Ec      WFDLFGMSMANGAHIAGLAVGLAMAFVDSLNARKRK 276
GlpG_Ss      WFDLFGMSMANGAHIAGLAVGLAMAFVDSLNARKRK 276
             ************************************

AarA_Ps      MAEQQNPFSIKSKARFSLGAIALTLTLVLLNIAVYFYQIVFASPLDSRESNLILFGANIY 60
Rhom7_Ss     -------MSASSVKPLNVQLPAITLILFALCIGIFCYLAQWMSYEEVDQSALIHLGANVA 53
                    :* .*   :.:   *:** *. * *.:: *   : *  :  :* ** :***:

AarA_Ps      QLSLTGDWWRYPISMMLHSNGTHLAFNCLALFVIGIGCERAYGKFKLLAIYIISGIGAAL 120
Rhom7_Ss     PLTLSGEPWRLLSSIFLHSSVSHLLMNMFAFLVVGGVAEQILGKWRLLITWLFSGVFGGL 113
              *:*:*: **   *::***. :** :* :*::*:*  .*:  **::**  :::**: ..*

AarA_Ps      FSAYWQYYEISNSDLWTDSTVYITIGVGASGAIMGIAAASVIYLIKVVINKPNPHPVIQR 180
Rhom7_Ss     ISACYALRE----------SEQIVISVGASGAILGIAGAAIATQFASG-------TGTYH 156
             :** :   *          :  *.*.*******:***.*::   :              :

AarA_Ps      RQKYQLYNLIAMIALTLING-LQSGVDNAAHIGGAIIGALISIAYILVPHKLRVAN-LCI 238
Rhom7_Ss     KNQRRVFPLLGMVALTLLYGARQTGIDNACHIGGLIAGGALGWLSARLSGQNRLVTEGGI 216
             ::: ::: *:.*:****: *  *:*:***.**** * *. :.     :  : *:..   *

AarA_Ps      TVIAASLLTMMIYLYSFSTNKHLLEEREFIYQEVYTELADANQ----------------- 281
Rhom7_Ss     IVAGSLLLTGAIWLAQQQMDESVLQVRQSLREAFYPQEIEQERRQKKQQLAEERNALRET 276
              * .: ***  *:* . . :: :*: *: : : .* :  : ::

AarA_Ps      ------------------------------------------------------------ 281
Rhom7_Ss     LSAPVSREQASGDLLAEIADIHDMAISRDGNMLYAAIENTNSIVVFDLGQKKILHTFTAP 336

AarA_Ps      ------------------------------------------------------------ 281
Rhom7_Ss     IAKEKSVKHCGGCKDQGVRSLALSPDEKLIYATSFEANALSVINVATGEIIQSITTGAHP 396

AarA_Ps      ------------------------------------------------------------ 281
Rhom7_Ss     DSLILSRDGTKAWVMNRTSNSVPAIDLVTYQHVADIPLEKYDGTGTSGKPGAWVMALSPD 456

AarA_Ps      ------------------------------------------------------------ 281
Rhom7_Ss     ERTLLVPGAGRGNIVRINTITHQKEDFPAGDARGTISAMRFRPENGEVIFADSQGISRIS 516

AarA_Ps      ------------------------------------------------------------ 281
Rhom7_Ss     VGDQQASIMTQWCSRSVYSVEGISPDGQYLALVSYGLQGYVILLNINAGQIIGVYPASYV 576

AarA_Ps      -------------------------------------------------  281
Rhom7_Ss     NHLRFSADGRKIFVMAKNGLIQMDRTRSLDPQAIIRHPQYGNVACIPEP  625
```

**Figure EV1. Amino acid sequence alignments of *S. sonnei* GlpG (GlpG_Ss) with *E. coli* GlpG (GlpG_Ec), and *S. sonnei* Rhom7 (Rhom7_Ss) with *P. stuartii* AarA (AarA_Ps).**
The single amino acid difference between the two versions of GlpG is highlighted by a purple arrow. The potential active site residues of Rhom7, Ser[133] and His[187], are highlighted by red arrows. Alignment performed by Clustal Omega.

 © 2020 The Authors

**Figure EV2. GlpG and Rhom7 do not influence the growth of *S. sonnei* under various conditions.**

A, B Wild-type *S. sonnei* or *S. sonnei* Δ*glpG*Δ*rhom7* were grown in liquid LB in the presence of oxygen at 37°C with shaking at 180 rpm. Growth was assessed by measuring the optical density of cultures at 600 nm ($OD_{600}$) (A) and colony-forming units of bacteria (CFU) recovered from the same samples (B).

C, D Bacteria were grown anaerobically in LB supplemented with 10 mM $KNO_3$ with shaking at 180 rpm. Growth was assessed by measuring the $OD_{600}$ as above (C) and CFU of bacteria recovered from the same cultures (D).

E, F Comparisons of cellular respiration (NADH production) of *S. sonnei* Δ*glpG*Δ*rhom7* (green) to wild-type *S. sonnei* (red) (E), or of *S. sonnei* Δ*glpG*Δ*rhom7* + *glpG* (green) to wild-type *S. sonnei* (red) (F) in conditions provided by PM1-10 of the Biolog MicroArrays. Areas of overlap are coloured yellow, while differences are shown as patches of red or green. Wild-type *S. sonnei* seemed to respire better than *S. sonnei* Δ*glpG*Δ*rhom7* + *glpG* in pH 9.5 + anthranilic acid (black box in E). However, this result was not reproducible.

Data information: In (A-D), data are presented as mean ± SD from three independent experiments.

© 2020 The Authors

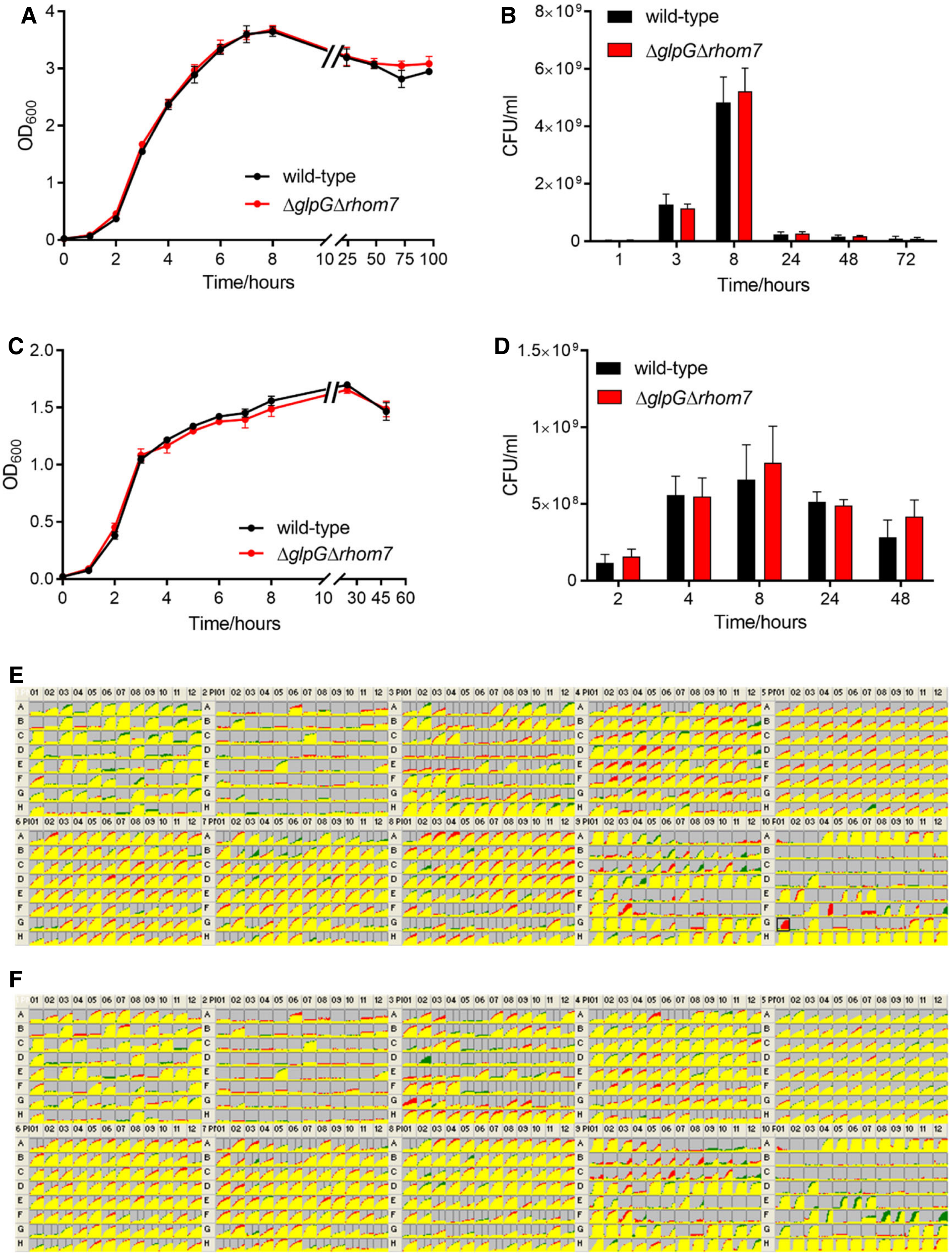


**Figure EV2.**

 © 2020 The Authors

 

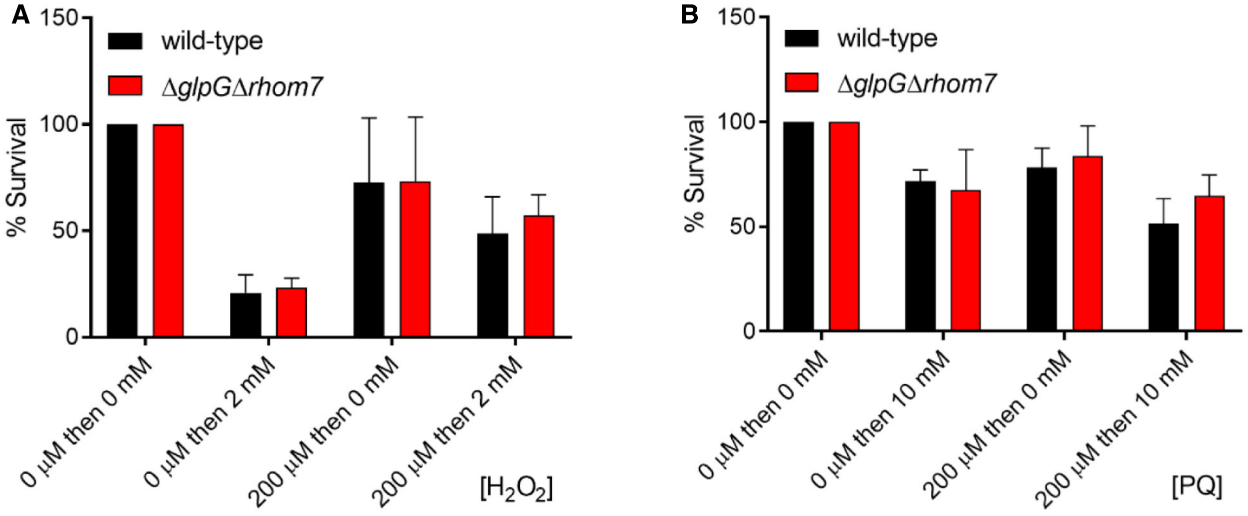


**Figure EV3. GlpG and Rhom7 do not affect *S. sonnei* survival following oxidative stress.**

A Resistance of wild-type *S. sonnei* or *S.sonnei ΔglpGΔrhom7* to $H_2O_2$. *S. sonnei* was grown in LB to an $OD_{600}$ of ≈ 0.5 and then pre-incubated in 0 or 200 μM $H_2O_2$ for 20 min, before incubation in 0 or 2 mM $H_2O_2$ for 15 min. Bacterial survival was measured by plating samples to solid media. The percentage survival was calculated as the ratio of CFU recovered from $H_2O_2$-exposed bacteria compared to bacteria not exposed to $H_2O_2$.

B Resistance of wild-type *S. sonnei* or *S. sonnei ΔglpGΔrhom7* to paraquat (PQ). *S. sonnei* was grown as above and then pre-incubated in 0 or 200 μM PQ for 15 min before incubation in 0 or 10 mM PQ for 45 min; samples were then plated to solid media to measure bacteria survival. The percentage survival was calculated as the ratio of CFU recovered from PQ-treated bacteria to those that were untreated.

Data information: In (A) and (B), data are presented as mean ± SD from three independent experiments.

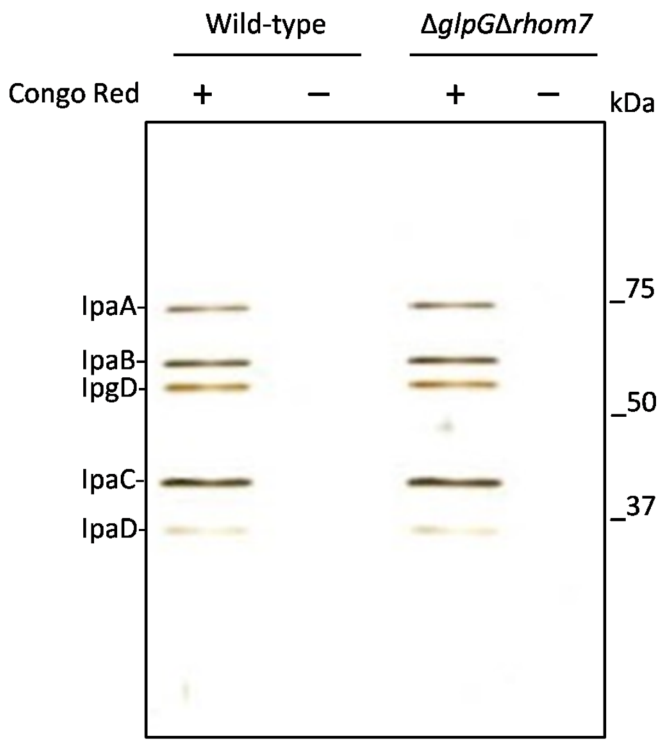


**Figure EV4. T3SS-mediated secretion of Ipas by *S. sonnei* is rhomboid-independent.**

T3SS-mediated secretion of effectors by wild-type *S. sonnei* and *S. sonnei ΔglpGΔrhom7*. Bacteria were grown to exponential phase ($OD_{600}$ ≈ 0.5), and Congo red (final concentration, 200 μg/ml) was added to bacteria to induce secretion. Supernatants were analysed by SDS–PAGE and silver staining. Sizes of a molecular weight marker are shown in kDa.

© 2020 The Authors 

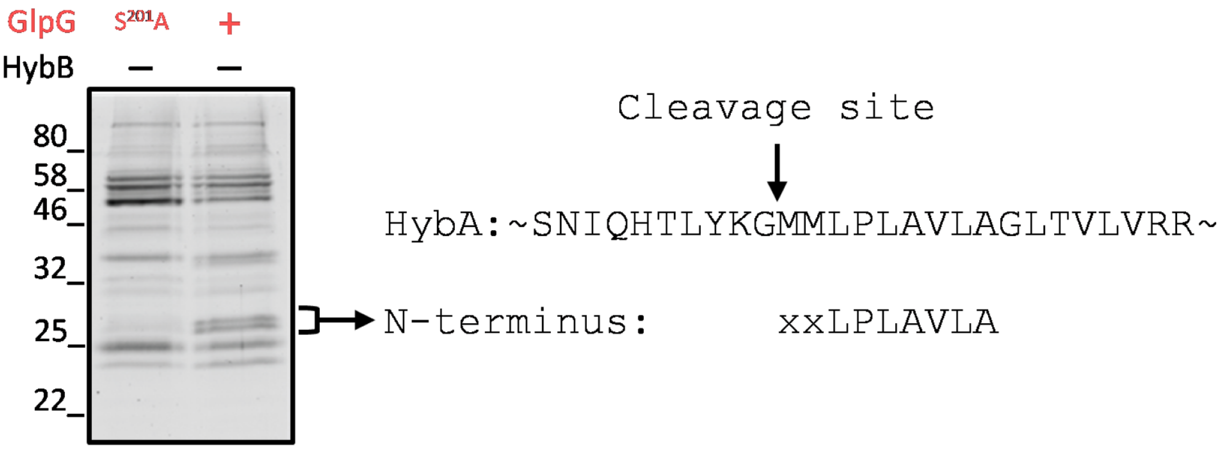


**Figure EV5. Identification of HybA cleavage site by GlpG.**
N-terminal sequencing of cleaved chromosomally encoded sfCherry-3xFLAG-tagged HybA by chromosomally encoded GlpG in *S. sonnei* in the absence of HybB. Bacteria were grown anaerobically in LB supplemented with 0.5% fumarate, 0.5% glycerol to $OD_{600} = 0.5$ before lysis. HybA was purified by anti-FLAG affinity chromatography prior to analysis by SDS–PAGE. Two bands only present in the GlpG (+) sample with molecular weight consistent with cleaved HybA were subject to N-terminal sequencing and gave the same result.

 © 2020 The Authors
