# Supplementary material for: Bacterial rhomboid proteases mediate quality control of orphan membrane proteins
Source: EMBO J. 2020 Apr 27;39(10):e102922. doi: 10.15252/embj.2019102922 (PMC7232013; doi:10.15252/embj.2019102922)
Supplement: Supplementary file 3 — Table EV1 [file EMBJ-39-e102922-s003.docx]

**Table EV1. Sequence of predicted TMDs and additional amino acids of putative rhomboid substrates from *S. sonnei* used as substrates for *in vivo* screening.**

| Putative substrate | Sequence inserted into artificial substrate. The predicted TMD is underlined. |
| --- | --- |
| CjrB | MNILHFSQSVKWSSWFICSLLLHGLIFLAFIWRFS |
| BcsB | GDVYYVGHLPWFERLWYALANHPILLAVLAAISVILLAWVLWRLL |
| CcmD | TPAFASWNEFFAMGGYAFFVWLAVVMTVIPLVVLVVHSVM |
| DgcJ | YVHDLPGGIRVSLSIDILYFITSSWKSVLFWILTALILLNMVRM |
| DjlA | QYWGKIIGVAVALLMGGGFWGVVLGLLIGHM |
| DjlB | ETVEPEPQPQPMPVDDGGLGCLGVIKIIFYIFIFAGLIGKILHLF |
| ElaB | YYYRAKQAVYRADDYVHEKPWQGIGVGAAVGLVLGLLLA |
| FdnH | LPKDPKIDTSVSLWKGALKPLAAAGFIATFAGLIFHYIGIG |
| FdoH | LPENPEISETVKFWKGIWKPLAAVGFAATFAASIFHYVGVGP |
| FliO | NNHATVQSSTPVSAAPLLQVSGALIAIIALILAAAWLV |
| Flk | NPFPPMMDTLQNMATRPALWILLVAIILMLVWLV |
| FtsH | LTKNVKVVGEPPEEPSLLASIFISWFPMLLLIGVWIFFMRQMQGG |
| HflK | GTGSGGGSSSQGPRPQLGGRVVTIAAAAIVIIWAASGFYTI |
| HyaA | SRVVDIPQMGTHSTADTVGLTALGVVAAAVGVHAVASAV |
| HybA | KLDDLSTGARSENIQHTLYKGMMLPLAVLAGLTVLVRR |
| HybO | TPRSQKPDVNAKEGGNVSAGAIGLLGGVVGLVAGVSVMAV |
| LapA | QQVTFNYLLAQGEYRISTLLAVLFAAGVAIGWLICGLFWLRV |
| MxiJ | YVYTNVQPVKEIKSEFLTNEVIYLFLGMAVLVVILLVWAFKTGWF |
| NrfF | TERYGDFVRYNPPLTGQTLVLWALPVVLLLLMALILWRV |
| PpdC | SASLKNQQGFSLPEVMVAMVLMVMIVTALSGIQ |
| RS17255 | SAQFWNRASIKQKIPLTILYILLFSVFLRLFFRHT |
| RsxB | NAIWIAVAAVSLLGLAFGAILGYAS |
| SecG | GASFGAGASATLFGSSGSGNFMTRMTALLATLFFIISLVLGNI |
| SohB | ELLSEYGLFLAKIVTVVLAIAAIAAIIVNVA |
| TcdA | VCAMKATAEGPKRMDCASGFGAATMVTATFGFVAVSHAL |
| TorS | NQHGLAHQEKASARGQYSLLLLGMVSLCALILILWRVVY |
| YajC | FFISDAVAATGAPAQGSPMSLILMLVVFGLIFYFMILR |
| YbdJ | RKHPGLFIGMYVAFFATLAVMLQSETLSGSVWLLVVLFILL |
| YbjT | SLEDKGDYRTIDVRAFWHPHGMPGLFYWLLMIPAHLFIFRG |
| YebO | NEVVNSGVMNIASLVVSVVVLLIGLILWFFI |
| YgaM | AARDAVGCADSFVRERPWCSVGTAAAVGIFIGALLSMR |
| YgiM | AQKKVDAASVQLDDKQRTIIMQWFMYGGGVLGLGLLLGLVLPHLI |
| YhcB | TWEYALIGLVVGIIIGAVAMRFGN |
| YhdP | VRRDLNMEAVVAPEISATVGVAAAFAVNPIVGAAVFAASKVL |
| YhhM | SKPPLFFIVIIGLIVVAASFRFM |
| YibN | QEIMQFVGRHPILSIAWIALLVAVLVTTFKSL |
| YjeT | MLYPKAWKKMISAMTNLPDNILRRFGGGLVVAGVVVYYMLRKTI |
| YmcD | TTTTGATTTATTTGGVAAGAVGTATVVGVATAVGVATLAVVA |
| YnaJ | EQYNIPLSEWTTSMYVIQSSMIFVYSLVFTVLLAIPLGIYFLGG |
| YqiK | DDIVNSVPSWMFTAIIAVCILFIIGIIFARLY |
| YqjD | TRVAAARADEYVRENPWTGVGIGAAIGVVLGVLLS |
| YtjB | DTHTLATEAQQVDNTTNILRLMLLLSLAIGVVLTRTLL |
| YtjC | NAALESCRDLPQGSRPLLVSHGIALGCLVSTILGLPAWA |
| ZipA | MQDLRLILIIVGAIAIIALLVHGFWTS |
