# Supplementary material for: Bacterial rhomboid proteases mediate quality control of orphan membrane proteins
Source: EMBO J. 2020 Apr 27;39(10):e102922. doi: 10.15252/embj.2019102922 (PMC7232013; doi:10.15252/embj.2019102922)
Supplement: Supplementary file 4 — Table EV2 [file EMBJ-39-e102922-s004.docx]

**Table EV2. Bacterial strains used in this study**

| **Strain ID** | **Reference** |
| --- | --- |
| DH5α, E. coli cloning strain | Lab collection |
| SM10 λpir | (Simon et al., 1983) |
| CS14, S. sonnei wild-type | (Holt et al., 2012) |
| CS14∆glpG∆rhom7 | This study |
| CS14∆glpG∆rhom7 glpG | This study |
| CS14∆glpG∆rhom7 pBAD33 glpG_WT_ | This study |
| CS14∆glpG∆rhom7 pBAD33 glpG_S201A_ | This study |
| CS14∆glpG∆rhom7 pBAD33 rhom7_WT_ | This study |
| CS14∆glpG∆rhom7 pBAD33 rhom7_S133A_ | This study |
| CS14∆glpG∆rhom7 pBAD33 rhom7_H187A_ | This study |
| CS14∆glpG∆rhom7 pBAD33 rhom7_ΔCTD_ | This study |
| CS14∆glpG∆rhom7 pBAD33 rhom7_ΔCTDΔTM7_ | This study |
| CS14 hybA-sfCherry-3xFLAG | This study |
| CS14∆glpG hybA-sfCherry-3xFLAG | This study |
| CS14∆rhom7 hybA-sfCherry-3xFLAG | This study |
| CS14∆glpG∆rhom7 hybA-sfCherry-3xFLAG | This study |
| CS14 hybO-sfCherry-3xFLAG | This study |
| CS14∆glpG∆rhom7 hybO-sfCherry-3xFLAG | This study |
| CS14∆glpG∆rhom7 hybO-sfCherry-3xFLAG pBAD33 3xHA-glpG_WT_ | This study |
| CS14∆glpG∆rhom7 hybO-sfCherry-3xFLAG pBAD33 3xHA-glpG_S201A H254A_ | This study |
| CS14∆glpG∆rhom7 hybO-sfCherry-3xFLAG pBAD33 rhom7_WT_ | This study |
| CS14∆glpG∆rhom7 hybO-sfCherry-3xFLAG pBAD33 rhom7_S133A H187A_ | This study |
| CS14∆glpG∆rhom7 hybA-sfCherry-3xFLAG pBAD33 3xHA-glpG_WT_ | This study |
| CS14∆glpG∆rhom7 hybA-sfCherry-3xFLAG pBAD33 3xHA-glpG_S201A H254A_ | This study |
| CS14∆glpG∆rhom7 hybA-sfCherry-3xFLAG pBAD33 rhom7_WT_ | This study |
| CS14∆glpG∆rhom7 hybA-sfCherry-3xFLAG pBAD33 rhom7_S133A H187A_ | This study |
| CS14 hybA_G296F_-sfCherry-3xFLAG | This study |
| CS14∆glpG∆rhom7 hybA_G296F_-sfCherry-3xFLAG | This study |
| CS14∆glpG∆rhom7 hybA_G296F_-sfCherry-3xFLAG pBAD33 3xHA-glpG_WT_ | This study |
| CS14∆glpG∆rhom7 hybA_G296F_-sfCherry-3xFLAG pBAD33 3xHA-glpG_S201A H254A_ | This study |
| CS14∆glpG∆rhom7 hybA_G296F_-sfCherry-3xFLAG pBAD33 rhom7_WT_ | This study |
| CS14∆glpG∆rhom7 hybA_G296F_-sfCherry-3xFLAG pBAD33 rhom7_S133A H187A_ | This study |
| CS14 hybA_P300A_-sfCherry-3xFLAG | This study |
| CS14∆glpG∆rhom7 hybA_P300A_-sfCherry-3xFLAG | This study |
| CS14∆glpG∆rhom7 hybA_P300A_-sfCherry-3xFLAG pBAD33 3xHA-glpG_WT_ | This study |
| CS14∆glpG∆rhom7 hybA_P300A_-sfCherry-3xFLAG pBAD33 3xHA-glpG_S201A H254A_ | This study |
| CS14∆glpG∆rhom7 hybA_P300A_-sfCherry-3xFLAG pBAD33 rhom7_WT_ | This study |
| CS14∆glpG∆rhom7 hybA_P300A_-sfCherry-3xFLAG pBAD33 rhom7_S133A H187A_  CS14∆rhom7 V5-hybA 3xHA-glpG_WT_  CS14∆rhom7 V5-hybA 3xHA-glpG_S201A_  CS14∆rhom7∆hybB V5-hybA 3xHA-glpG_WT_  CS14∆rhom7∆hybB V5-hybA 3xHA-glpG_S201A_ | This study  This study  This study  This study  This study |
| CS14∆rhom7∆cyaA 3xHA-glpG_WT_ | This study |
| CS14∆rhom7∆cyaA hybA-T25-3xFLAG 3xHA-glpG_WT_ | This study |
| CS14∆rhom7∆cyaA hybB-His-T18 3xHA-glpG_WT_ | This study |
| CS14∆rhom7∆cyaA hybA-T25-3xFLAG hybB-His-T18 3xHA-glpG_WT_ | This study |
| CS14∆rhom7 hybA-sfCherry-3xFLAG 3xHA-glpG_WT_ | This study |
| CS14∆rhom7 hybA-sfCherry-3xFLAG 3xHA-glpG_S201A_ | This study |
| CS14∆rhom7∆hybB hybA-*sfCherry-3xFLAG* 3xHA-glpG_WT_ | This study |
| CS14∆rhom7∆hybB hybA-*sfCherry-3xFLAG* 3xHA-glpG_S201A_ | This study |
| CS14∆rhom7∆hybB hybA_P300A_-sfCherry-3xFLAG 3xHA-glpG_WT_ | This study |
| CS14∆rhom7∆hybB hybA_P300A_-sfCherry-3xFLAG 3xHA-glpG_S201A_ | This study |
| CS14∆rhom7 hybA*-sfCherry-3xFLAG* 3xHA-glpG_WT_ pKS508 hybA-*sfCherry-3xFLAG* | This study |
| CS14∆rhom7 hybA-*sfCherry-3xFLAG* 3xHA-glpG_S201A_ pKS508 hybA-*sfCherry-3xFLAG* | This study |
| CS14∆rhom7 hybA-*sfCherry-3xFLAG* 3xHA-glpG_WT_ pUC19 3xHA-glpG_WT_ | This study |
| CS14∆rhom7 hybA-*sfCherry-3xFLAG* 3xHA-glpG_S201A_ pUC19 3xHA-glpG_S201A H254A_ | This study |
| CS14∆hyaA-F∆hycE∆spa33 | This study |
| CS14∆hyaA-F∆hybO-G∆hycE∆spa33 | This study |
| CS14∆hyaA-F∆hycE∆spa33∆rhom7 | This study |
| CS14∆hyaA-F∆hycE∆spa33∆glpG∆rhom7 | This study |
| CS14∆hyaA-F∆hycE∆spa33∆rhom7 hybA_G296F_ | This study |
| CS14∆hyaA-F∆hycE∆spa33∆rhom7 hybA-*sfCherry-3xFLAG* | This study |
| CS14∆hyaA-F∆hycE∆spa33∆glpG∆rhom7 pUC19 3xHA-glpG_WT_ | This study |
| CS14∆hyaA-F∆hycE∆spa33∆glpG∆rhom7 pUC19 3xHA-glpG_S201A H254A_ | This study |
| CS14∆rhom7 fdoH-*sfCherry-3xFLAG* 3xHA-glpG_WT_ | This study |
| CS14∆rhom7 fdoH-*sfCherry-3xFLAG* 3xHA-glpG_S201A_ | This study |
| CS14∆rhom7∆fdoI fdoH-*sfCherry-3xFLAG* 3xHA-glpG_WT_ | This study |
| CS14∆rhom7∆fdoI fdoH-*sfCherry-3xFLAG* 3xHA-glpG_S201A_ | This study |
| CS14∆rhom7 fdoH_P259A_-*sfCherry-3xFLAG* 3xHA-glpG_WT_ | This study |
| CS14∆rhom7 fdoH_P259A_-*sfCherry-3xFLAG* 3xHA-glpG_S201A_ | This study |
| CS14∆rhom7∆fdoI fdoH_P259A_-*sfCherry-3xFLAG* 3xHA-glpG_WT_ | This study |
| CS14∆rhom7∆fdoI fdoH_P259A_-*sfCherry-3xFLAG* 3xHA-glpG_S201A_ | This study |
| CS14∆glpG fdnH-*sfCherry-3xFLAG* | This study |
| CS14∆glpG∆rhom7 fdnH-*sfCherry-3xFLAG* | This study |
| CS14∆glpG∆fdnI fdnH-*sfCherry-3xFLAG* | This study |
| CS14∆glpG∆rhom7∆fdnI fdnH-*sfCherry-3xFLAG* | This study |
| CS14∆glpG∆rhom7 fdnH-*sfCherry-3xFLAG* pBAD33 rhom7_WT_ | This study |
| CS14∆glpG∆rhom7 fdnH-*sfCherry-3xFLAG* pBAD33 rhom7_S133A H187A_ | This study |
| CS14∆glpG∆rhom7∆fdnI fdnH-*sfCherry-3xFLAG* pBAD33 rhom7_WT_ | This study |
| CS14∆glpG∆rhom7∆fdnI fdnH-*sfCherry-3xFLAG* pBAD33 rhom7_S133A H187A_ | This study |
| CS14∆glpG∆rhom7 fdnH_P259A_-*sfCherry-3xFLAG* pBAD33 rhom7_WT_ | This study |
| CS14∆glpG∆rhom7 fdnH_P259A_-*sfCherry-3xFLAG* pBAD33 rhom7_S133A H187A_ | This study |
| CS14∆glpG∆rhom7∆fdnI fdnH_P259A_-*sfCherry-3xFLAG* pBAD33 rhom7_WT_ | This study |
| CS14∆glpG∆rhom7∆fdnI fdnH_P259A_-*sfCherry-3xFLAG* pBAD33 rhom7_S133A H187A_ | This study |
| CS14∆glpG∆rhom7 pBAD33 3xHA-glpG_WT_ pKS508 | This study |
| CS14∆glpG∆rhom7 pBAD33 3xHA-glpG_S201A H254A_ pKS508 | This study |
| CS14∆glpG∆rhom7 pBAD33 3xHA-glpG_WT_ pKS508 CjrB | This study |
| CS14∆glpG∆rhom7 pBAD33 3xHA-glpG_S201A H254A_ pKS508 CjrB | This study |
| CS14∆glpG∆rhom7 pBAD33 3xHA-glpG_WT_ pKS508 BcsB | This study |
| CS14∆glpG∆rhom7 pBAD33 3xHA-glpG_S201A H254A_ pKS508 BcsB | This study |
| CS14∆glpG∆rhom7 pBAD33 3xHA-glpG_WT_ pKS508 CcmD | This study |
| CS14∆glpG∆rhom7 pBAD33 3xHA-glpG_S201A H254A_ pKS508 CcmD | This study |
| CS14∆glpG∆rhom7 pBAD33 3xHA-glpG_WT_ pKS508 DgcJ | This study |
| CS14∆glpG∆rhom7 pBAD33 3xHA-glpG_S201A H254A_ pKS508 DgcJ | This study |
| CS14∆glpG∆rhom7 pBAD33 3xHA-glpG_WT_ pKS508 DjlA | This study |
| CS14∆glpG∆rhom7 pBAD33 3xHA-glpG_S201A H254A_ pKS508 DjlA | This study |
| CS14∆glpG∆rhom7 pBAD33 3xHA-glpG_WT_ pKS508 DjlB | This study |
| CS14∆glpG∆rhom7 pBAD33 3xHA-glpG_S201A H254A_ pKS508 DjlB | This study |
| CS14∆glpG∆rhom7 pBAD33 3xHA-glpG_WT_ pKS508 ElaB | This study |
| CS14∆glpG∆rhom7 pBAD33 3xHA-glpG_S201A H254A_ pKS508 ElaB | This study |
| CS14∆glpG∆rhom7 pBAD33 3xHA-glpG_WT_ pKS508 FdnH | This study |
| CS14∆glpG∆rhom7 pBAD33 3xHA-glpG_S201A H254A_ pKS508 FdnH | This study |
| CS14∆glpG∆rhom7 pBAD33 3xHA-glpG_WT_ pKS508 FdoH | This study |
| CS14∆glpG∆rhom7 pBAD33 3xHA-glpG_S201A H254A_ pKS508 FdoH | This study |
| CS14∆glpG∆rhom7 pBAD33 3xHA-glpG_WT_ pKS508 YebO | This study |
| CS14∆glpG∆rhom7 pBAD33 3xHA-glpG_S201A H254A_ pKS508 YebO | This study |
| CS14∆glpG∆rhom7 pBAD33 3xHA-glpG_WT_ pKS508 FliO | This study |
| CS14∆glpG∆rhom7 pBAD33 3xHA-glpG_S201A H254A_ pKS508 FliO | This study |
| CS14∆glpG∆rhom7 pBAD33 3xHA-glpG_WT_ pKS508 Flk | This study |
| CS14∆glpG∆rhom7 pBAD33 3xHA-glpG_S201A H254A_ pKS508 Flk | This study |
| CS14∆glpG∆rhom7 pBAD33 3xHA-glpG_WT_ pKS508 FtsH | This study |
| CS14∆glpG∆rhom7 pBAD33 3xHA-glpG_S201A H254A_ pKS508 FtsH | This study |
| CS14∆glpG∆rhom7 pBAD33 3xHA-glpG_WT_ pKS508 HflK | This study |
| CS14∆glpG∆rhom7 pBAD33 3xHA-glpG_S201A H254A_ pKS508 HflK | This study |
| CS14∆glpG∆rhom7 pBAD33 3xHA-glpG_WT_ pKS508 HyaA | This study |
| CS14∆glpG∆rhom7 pBAD33 3xHA-glpG_S201A H254A_ pKS508 HyaA | This study |
| CS14∆glpG∆rhom7 pBAD33 3xHA-glpG_WT_ pKS508 HybA | This study |
| CS14∆glpG∆rhom7 pBAD33 3xHA-glpG_S201A H254A_ pKS508 HybA | This study |
| CS14∆glpG∆rhom7 pBAD33 3xHA-glpG_WT_ pKS508 HybO | This study |
| CS14∆glpG∆rhom7 pBAD33 3xHA-glpG_S201A H254A_ pKS508 HybO | This study |
| CS14∆glpG∆rhom7 pBAD33 3xHA-glpG_WT_ pKS508 LapA | This study |
| CS14∆glpG∆rhom7 pBAD33 3xHA-glpG_S201A H254A_ pKS508 LapA | This study |
| CS14∆glpG∆rhom7 pBAD33 3xHA-glpG_WT_ pKS508 MxiJ | This study |
| CS14∆glpG∆rhom7 pBAD33 3xHA-glpG_S201A H254A_ pKS508 MxiJ | This study |
| CS14∆glpG∆rhom7 pBAD33 3xHA-glpG_WT_ pKS508 NrfF | This study |
| CS14∆glpG∆rhom7 pBAD33 3xHA-glpG_S201A H254A_ pKS508 NrfF | This study |
| CS14∆glpG∆rhom7 pBAD33 3xHA-glpG_WT_ pKS508 PpdC | This study |
| CS14∆glpG∆rhom7 pBAD33 3xHA-glpG_S201A H254A_ pKS508 PpdC | This study |
| CS14∆glpG∆rhom7 pBAD33 3xHA-glpG_WT_ pKS508 RS17255 | This study |
| CS14∆glpG∆rhom7 pBAD33 3xHA-glpG_S201A H254A_ pKS508 RS17255 | This study |
| CS14∆glpG∆rhom7 pBAD33 3xHA-glpG_WT_ pKS508 RxsB | This study |
| CS14∆glpG∆rhom7 pBAD33 3xHA-glpG_S201A H254A_ pKS508 RxsB | This study |
| CS14∆glpG∆rhom7 pBAD33 3xHA-glpG_WT_ pKS508 SecG | This study |
| CS14∆glpG∆rhom7 pBAD33 3xHA-glpG_S201A H254A_ pKS508 SecG | This study |
| CS14∆glpG∆rhom7 pBAD33 3xHA-glpG_WT_ pKS508 SohB | This study |
| CS14∆glpG∆rhom7 pBAD33 3xHA-glpG_S201A H254A_ pKS508 SohB | This study |
| CS14∆glpG∆rhom7 pBAD33 3xHA-glpG_WT_ pKS508 TcdA | This study |
| CS14∆glpG∆rhom7 pBAD33 3xHA-glpG_S201A H254A_ pKS508 TcdA | This study |
| CS14∆glpG∆rhom7 pBAD33 3xHA-glpG_WT_ pKS508 TorS | This study |
| CS14∆glpG∆rhom7 pBAD33 3xHA-glpG_S201A H254A_ pKS508 TorS | This study |
| CS14∆glpG∆rhom7 pBAD33 3xHA-glpG_WT_ pKS508 YajC | This study |
| CS14∆glpG∆rhom7 pBAD33 3xHA-glpG_S201A H254A_ pKS508 YajC | This study |
| CS14∆glpG∆rhom7 pBAD33 3xHA-glpG_WT_ pKS508 YbdJ | This study |
| CS14∆glpG∆rhom7 pBAD33 3xHA-glpG_S201A H254A_ pKS508 YbdJ | This study |
| CS14∆glpG∆rhom7 pBAD33 3xHA-glpG_WT_ pKS508 YbjT | This study |
| CS14∆glpG∆rhom7 pBAD33 3xHA-glpG_S201A H254A_ pKS508 YbjT | This study |
| CS14∆glpG∆rhom7 pBAD33 3xHA-glpG_WT_ pKS508 YgaM | This study |
| CS14∆glpG∆rhom7 pBAD33 3xHA-glpG_S201A H254A_ pKS508 YgaM | This study |
| CS14∆glpG∆rhom7 pBAD33 3xHA-glpG_WT_ pKS508 YgiM | This study |
| CS14∆glpG∆rhom7 pBAD33 3xHA-glpG_S201A H254A_ pKS508 YgiM | This study |
| CS14∆glpG∆rhom7 pBAD33 3xHA-glpG_WT_ pKS508 YhcB | This study |
| CS14∆glpG∆rhom7 pBAD33 3xHA-glpG_S201A H254A_ pKS508 YhcB | This study |
| CS14∆glpG∆rhom7 pBAD33 3xHA-glpG_WT_ pKS508 YhdP | This study |
| CS14∆glpG∆rhom7 pBAD33 3xHA-glpG_S201A H254A_ pKS508 YhdP | This study |
| CS14∆glpG∆rhom7 pBAD33 3xHA-glpG_WT_ pKS508 YhhM | This study |
| CS14∆glpG∆rhom7 pBAD33 3xHA-glpG_S201A H254A_ pKS508 YhhM | This study |
| CS14∆glpG∆rhom7 pBAD33 3xHA-glpG_WT_ pKS508 YibN | This study |
| CS14∆glpG∆rhom7 pBAD33 3xHA-glpG_S201A H254A_ pKS508 YibN | This study |
| CS14∆glpG∆rhom7 pBAD33 3xHA-glpG_WT_ pKS508 YjeT | This study |
| CS14∆glpG∆rhom7 pBAD33 3xHA-glpG_S201A H254A_ pKS508 YjeT | This study |
| CS14∆glpG∆rhom7 pBAD33 3xHA-glpG_WT_ pKS508 YmcD | This study |
| CS14∆glpG∆rhom7 pBAD33 3xHA-glpG_S201A H254A_ pKS508 YmcD | This study |
| CS14∆glpG∆rhom7 pBAD33 3xHA-glpG_WT_ pKS508 YnaJ | This study |
| CS14∆glpG∆rhom7 pBAD33 3xHA-glpG_S201A H254A_ pKS508 YnaJ | This study |
| CS14∆glpG∆rhom7 pBAD33 3xHA-glpG_WT_ pKS508 YqiK | This study |
| CS14∆glpG∆rhom7 pBAD33 3xHA-glpG_S201A H254A_ pKS508 YqiK | This study |
| CS14∆glpG∆rhom7 pBAD33 3xHA-glpG_WT_ pKS508 YqjD | This study |
| CS14∆glpG∆rhom7 pBAD33 3xHA-glpG_S201A H254A_ pKS508 YqjD | This study |
| CS14∆glpG∆rhom7 pBAD33 3xHA-glpG_WT_ pKS508 YtjB | This study |
| CS14∆glpG∆rhom7 pBAD33 3xHA-glpG_S201A H254A_ pKS508 YtjB | This study |
| CS14∆glpG∆rhom7 pBAD33 3xHA-glpG_WT_ pKS508 YtjC | This study |
| CS14∆glpG∆rhom7 pBAD33 3xHA-glpG_S201A H254A_ pKS508 YtjC | This study |
| CS14∆glpG∆rhom7 pBAD33 3xHA-glpG_WT_ pKS508 ZipA | This study |
| CS14∆glpG∆rhom7 pBAD33 3xHA-glpG_S201A H254A_ pKS508 ZipA | This study |
| CS14∆glpG∆rhom7 pBAD33 3xHA-glpG_WT_ pKS508 YibN | This study |
| CS14∆glpG∆rhom7 pBAD33 3xHA-glpG_S201A H254A_ pKS508 YibN | This study |
| CS14∆glpG∆rhom7 pBAD33 3xHA-glpG_WT_ pKS508 YjeT | This study |
| CS14∆glpG∆rhom7 pBAD33 3xHA-glpG_S201A H254A_ pKS508 YjeT | This study |
| CS14∆glpG∆rhom7 pBAD33 3xHA-rhom7_WT_ pKS508 | This study |
| CS14∆glpG∆rhom7 pBAD33 3xHA-rhom7_S133A H187A_ pKS508 | This study |
| CS14∆glpG∆rhom7 pBAD33 3xHA-rhom7_WT_ pKS508 CjrB | This study |
| CS14∆glpG∆rhom7 pBAD33 3xHA-rhom7_S133A H187A_ pKS508 CjrB | This study |
| CS14∆glpG∆rhom7 pBAD33 3xHA-rhom7_WT_ pKS508 BcsB | This study |
| CS14∆glpG∆rhom7 pBAD33 3xHA-rhom7_S133A H187A_ pKS508 BcsB | This study |
| CS14∆glpG∆rhom7 pBAD33 3xHA-rhom7_WT_ pKS508 CcmD | This study |
| CS14∆glpG∆rhom7 pBAD33 3xHA-rhom7_S133A H187A_ pKS508 CcmD | This study |
| CS14∆glpG∆rhom7 pBAD33 3xHA-rhom7_WT_ pKS508 DgcJ | This study |
| CS14∆glpG∆rhom7 pBAD33 3xHA-rhom7_S133A H187A_ pKS508 DgcJ | This study |
| CS14∆glpG∆rhom7 pBAD33 3xHA-rhom7_WT_ pKS508 DjlA | This study |
| CS14∆glpG∆rhom7 pBAD33 3xHA-rhom7_S133A H187A_ pKS508 DjlA | This study |
| CS14∆glpG∆rhom7 pBAD33 3xHA-rhom7_WT_ pKS508 DjlB | This study |
| CS14∆glpG∆rhom7 pBAD33 3xHA-rhom7_S133A H187A_ pKS508 DjlB | This study |
| CS14∆glpG∆rhom7 pBAD33 3xHA-rhom7_WT_ pKS508 ElaB | This study |
| CS14∆glpG∆rhom7 pBAD33 3xHA-rhom7_S133A H187A_ pKS508 ElaB | This study |
| CS14∆glpG∆rhom7 pBAD33 rhom7_WT_ pKS508 FdnH | This study |
| CS14∆glpG∆rhom7 pBAD33 rhom7_S133A H187A_ pKS508 FdnH | This study |
| CS14∆glpG∆rhom7 pBAD33 rhom7_WT_ pKS508 FdoH | This study |
| CS14∆glpG∆rhom7 pBAD33 rhom7_S133A H187A_ pKS508 FdoH | This study |
| CS14∆glpG∆rhom7 pBAD33 3xHA-rhom7_WT_ pKS508 YebO | This study |
| CS14∆glpG∆rhom7 pBAD33 3xHA-rhom7_S133A H187A_ pKS508 YebO | This study |
| CS14∆glpG∆rhom7 pBAD33 3xHA-rhom7_WT_ pKS508 FliO | This study |
| CS14∆glpG∆rhom7 pBAD33 3xHA-rhom7_S133A H187A_ pKS508 FliO | This study |
| CS14∆glpG∆rhom7 pBAD33 3xHA-rhom7_WT_ pKS508 Flk | This study |
| CS14∆glpG∆rhom7 pBAD33 3xHA-rhom7_S133A H187A_ pKS508 Flk | This study |
| CS14∆glpG∆rhom7 pBAD33 3xHA-rhom7_WT_ pKS508 FtsH | This study |
| CS14∆glpG∆rhom7 pBAD33 3xHA-rhom7_S133A H187A_ pKS508 FtsH | This study |
| CS14∆glpG∆rhom7 pBAD33 3xHA-rhom7_WT_ pKS508 HflK | This study |
| CS14∆glpG∆rhom7 pBAD33 3xHA-rhom7_S133A H187A_ pKS508 HflK | This study |
| CS14∆glpG∆rhom7 pBAD33 3xHA-rhom7_WT_ pKS508 HyaA | This study |
| CS14∆glpG∆rhom7 pBAD33 3xHA-rhom7_S133A H187A_ pKS508 HyaA | This study |
| CS14∆glpG∆rhom7 pBAD33 3xHA-rhom7_WT_ pKS508 HybA | This study |
| CS14∆glpG∆rhom7 pBAD33 3xHA-rhom7_S133A H187A_ pKS508 HybA | This study |
| CS14∆glpG∆rhom7 pBAD33 3xHA-rhom7_WT_ pKS508 HybO | This study |
| CS14∆glpG∆rhom7 pBAD33 3xHA-rhom7_S133A H187A_ pKS508 HybO | This study |
| CS14∆glpG∆rhom7 pBAD33 3xHA-rhom7_WT_ pKS508 LapA | This study |
| CS14∆glpG∆rhom7 pBAD33 3xHA-rhom7_S133A H187A_ pKS508 LapA | This study |
| CS14∆glpG∆rhom7 pBAD33 3xHA-rhom7_WT_ pKS508 MxiJ | This study |
| CS14∆glpG∆rhom7 pBAD33 3xHA-rhom7_S133A H187A_ pKS508 MxiJ | This study |
| CS14∆glpG∆rhom7 pBAD33 3xHA-rhom7_WT_ pKS508 NrfF | This study |
| CS14∆glpG∆rhom7 pBAD33 3xHA-rhom7_S133A H187A_ pKS508 NrfF | This study |
| CS14∆glpG∆rhom7 pBAD33 3xHA-rhom7_WT_ pKS508 PpdC | This study |
| CS14∆glpG∆rhom7 pBAD33 3xHA-rhom7_S133A H187A_ pKS508 PpdC | This study |
| CS14∆glpG∆rhom7 pBAD33 3xHA-rhom7_WT_ pKS508 RS17255 | This study |
| CS14∆glpG∆rhom7 pBAD33 rhom7_S133A H187A_ pKS508 RS17255 | This study |
| CS14∆glpG∆rhom7 pBAD33 rhom7_WT_ pKS508 RxsB | This study |
| CS14∆glpG∆rhom7 pBAD33 3xHA-rhom7_S133A H187A_ pKS508 RxsB | This study |
| CS14∆glpG∆rhom7 pBAD33 3xHA-rhom7_WT_ pKS508 SecG | This study |
| CS14∆glpG∆rhom7 pBAD33 3xHA-rhom7_S133A H187A_ pKS508 SecG | This study |
| CS14∆glpG∆rhom7 pBAD33 rhom7_WT_ pKS508 SohB | This study |
| CS14∆glpG∆rhom7 pBAD33 rhom7_S133A H187A_ pKS508 SohB | This study |
| CS14∆glpG∆rhom7 pBAD33 3xHA-rhom7_WT_ pKS508 TcdA | This study |
| CS14∆glpG∆rhom7 pBAD33 3xHA-rhom7_S133A H187A_ pKS508 TcdA | This study |
| CS14∆glpG∆rhom7 pBAD33 rhom7_WT_ pKS508 TorS | This study |
| CS14∆glpG∆rhom7 pBAD33 rhom7_S133A H187A_ pKS508 TorS | This study |
| CS14∆glpG∆rhom7 pBAD33 3xHA-rhom7_WT_ pKS508 YajC | This study |
| CS14∆glpG∆rhom7 pBAD33 3xHA-rhom7_S133A H187A_ pKS508 YajC | This study |
| CS14∆glpG∆rhom7 pBAD33 3xHA-rhom7_WT_ pKS508 YbdJ | This study |
| CS14∆glpG∆rhom7 pBAD33 3xHA-rhom7_S133A H187A_ pKS508 YbdJ | This study |
| CS14∆glpG∆rhom7 pBAD33 3xHA-rhom7_WT_ pKS508 YbjT | This study |
| CS14∆glpG∆rhom7 pBAD33 3xHA-rhom7_S133A H187A_ pKS508 YbjT | This study |
| CS14∆glpG∆rhom7 pBAD33 3xHA-rhom7_WT_ pKS508 YgaM | This study |
| CS14∆glpG∆rhom7 pBAD33 3xHA-rhom7_S133A H187A_ pKS508 YgaM | This study |
| CS14∆glpG∆rhom7 pBAD33 3xHA-rhom7_WT_ pKS508 YgiM | This study |
| CS14∆glpG∆rhom7 pBAD33 3xHA-rhom7_S133A H187A_ pKS508 YgiM | This study |
| CS14∆glpG∆rhom7 pBAD33 3xHA-rhom7_WT_ pKS508 YhcB | This study |
| CS14∆glpG∆rhom7 pBAD33 3xHA-rhom7_S133A H187A_ pKS508 YhcB | This study |
| CS14∆glpG∆rhom7 pBAD33 3xHA-rhom7_WT_ pKS508 YhdP | This study |
| CS14∆glpG∆rhom7 pBAD33 3xHA-rhom7_S133A H187A_ pKS508 YhdP | This study |
| CS14∆glpG∆rhom7 pBAD33 3xHA-rhom7_WT_ pKS508 YhhM | This study |
| CS14∆glpG∆rhom7 pBAD33 3xHA-rhom7_S133A H187A_ pKS508 YhhM | This study |
| CS14∆glpG∆rhom7 pBAD33 3xHA-rhom7_WT_ pKS508 YibN | This study |
| CS14∆glpG∆rhom7 pBAD33 3xHA-rhom7_S133A H187A_ pKS508 YibN | This study |
| CS14∆glpG∆rhom7 pBAD33 3xHA-rhom7_WT_ pKS508 YjeT | This study |
| CS14∆glpG∆rhom7 pBAD33 3xHA-rhom7_S133A H187A_ pKS508 YjeT | This study |
| CS14∆glpG∆rhom7 pBAD33 3xHA-rhom7_WT_ pKS508 YmcD | This study |
| CS14∆glpG∆rhom7 pBAD33 3xHA-rhom7_S133A H187A_ pKS508 YmcD | This study |
| CS14∆glpG∆rhom7 pBAD33 3xHA-rhom7_WT_ pKS508 YnaJ | This study |
| CS14∆glpG∆rhom7 pBAD33 3xHA-rhom7_S133A H187A_ pKS508 YnaJ | This study |
| CS14∆glpG∆rhom7 pBAD33 3xHA-rhom7_WT_ pKS508 YqiK | This study |
| CS14∆glpG∆rhom7 pBAD33 3xHA-rhom7_S133A H187A_ pKS508 YqiK | This study |
| CS14∆glpG∆rhom7 pBAD33 3xHA-rhom7_WT_ pKS508 YqjD | This study |
| CS14∆glpG∆rhom7 pBAD33 3xHA-rhom7_S133A H187A_ pKS508 YqjD | This study |
| CS14∆glpG∆rhom7 pBAD33 3xHA-rhom7_WT_ pKS508 YtjB | This study |
| CS14∆glpG∆rhom7 pBAD33 3xHA-rhom7_S133A H187A_ pKS508 YtjB | This study |
| CS14∆glpG∆rhom7 pBAD33 3xHA-rhom7_WT_ pKS508 YtjC | This study |
| CS14∆glpG∆rhom7 pBAD33 3xHA-rhom7_S133A H187A_ pKS508 YtjC | This study |
| CS14∆glpG∆rhom7 pBAD33 3xHA-rhom7_WT_ pKS508 ZipA | This study |
| CS14∆glpG∆rhom7 pBAD33 3xHA-rhom7_S133A H187A_ pKS508 ZipA | This study |
| CS14∆glpG∆rhom7 pBAD33 3xHA-rhom7_WT_ pKS508 YibN | This study |
| CS14∆glpG∆rhom7 pBAD33 3xHA-rhom7_S133A H187A_ pKS508 YibN | This study |
| CS14∆glpG∆rhom7 pBAD33 3xHA-rhom7_WT_ pKS508 YjeT | This study |
| CS14∆glpG∆rhom7 pBAD33 3xHA-rhom7_S133A H187A_ pKS508 YjeT | This study |
