# Supplementary material for: Bacterial rhomboid proteases mediate quality control of orphan membrane proteins
Source: EMBO J. 2020 Apr 27;39(10):e102922. doi: 10.15252/embj.2019102922 (PMC7232013; doi:10.15252/embj.2019102922)
Supplement: Supplementary file 5 — Table EV3 [file EMBJ-39-e102922-s005.docx]

**Table EV3. Plasmids used in this study**

| **Plasmid name** | **Reference** |
| --- | --- |
| pBAD33 | (Guzman et al., 1995) |
| pBAD33 *3xHA-glpG* | This study |
| pBAD33 *3xHA-glpG*_S201A_ | This study |
| pBAD33 *3xHA-glpG*_S201A H254A_ | This study |
| pBAD33 *rhom7* | This study |
| pBAD33 *rhom7*_S133A_ | This study |
| pBAD33 *3xHA-rhom7* | This study |
| pBAD33 *3xHA-rhom7*_S133A_ | This study |
| pBAD33 *rhom7*_S133A H187A_ | This study |
| pBAD33 *3xHA-rhom7*_∆TM7_ | This study |
| pBAD33 *3xHA-rhom7*_∆TM7∆CTD_ | This study |
| pUC19 | (Yanisch-Perron et al., 1985) |
| pUC19 *3xHA-glpG* | This study |
| pUC19 *3xHA-glpG*_S201A H254A_ | This study |
| pLAC101 *V5-hybA* | This study |
| pLAC101 *V5-hybA*_P300A_ | This study |
| pKS508 | (Strisovsky et al., 2009) |
| pKS508 TM-CjrB | This study |
| pKS508 TM-BcsB | This study |
| pKS508 TM-CcmD | This study |
| pKS508 TM-DgcJ | This study |
| pKS508 TM-DjlA | This study |
| pKS508 TM-DjlB | This study |
| pKS508 TM-ElaB | This study |
| pKS508 TM-FdnH | This study |
| pKS508 TM-FdoH | This study |
| pKS508 TM-YebO | This study |
| pKS508 TM-FliO | This study |
| pKS508 TM-Flk | This study |
| pKS508 TM-FtsH | This study |
| pKS508 TM-HflK | This study |
| pKS508 TM-HyaA | This study |
| pKS508 TM-HybA | This study |
| pKS508 TM-HybO | This study |
| pKS508 TM-LapA | This study |
| pKS508 TM-MxiJ | This study |
| pKS508 TM-NrfF | This study |
| pKS508 TM-PpdC | This study |
| pKS508 TM-RS17255 | This study |
| pKS508 TM-RxsB | This study |
| pKS508 TM-SecG | This study |
| pKS508 TM-SohB | This study |
| pKS508 TM-TcdA | This study |
| pKS508 TM-TorS | This study |
| pKS508 TM-YajC | This study |
| pKS508 TM-YbdJ | This study |
| pKS508 TM-YbjT | This study |
| pKS508 TM-YgaM | This study |
| pKS508 TM-YgiM | This study |
| pKS508 TM-YhcB | This study |
| pKS508 TM-YhdP | This study |
| pKS508 TM-YhhM | This study |
| pKS508 TM-YibN | This study |
| pKS508 TM-YjeT | This study |
| pKS508 TM-YmcD | This study |
| pKS508 TM-YnaJ | This study |
| pKS508 TM-YqiK | This study |
| pKS508 TM-YqjD | This study |
| pKS508 TM-YtjB | This study |
| pKS508 TM-YtjC | This study |
| pKS508 TM-ZipA | This study |
| pKS508 *hybA-sfCherry-3xFLAG* | This study |
| pKNG101 | (Kaniga et al., 1991) |
| pCONJ4s | This study |
| pCONJ4s ∆*glpG* | This study |
| pCONJ4s *glpG*_S201A_ | This study |
| pCONJ4s *3xHA-glpG*  pCONJ4s *V5-hybA* | This study  This study |
| pCONJ4s ∆*rhom7* | This study |
| pCONJ4s ∆*spa33* | This study |
| pCONJ4s ∆*hyaA-F* | This study |
| pCONJ4s ∆*hybO-G* | This study |
| pCONJ4s ∆*hycE* | This study |
| pCONJ4s ∆*hybB* (*hybA-sfCherry-3xFLAG*) | This study |
| pCONJ4s ∆*hybB* (*V5-hybA*) | This study |
| pCONJ4s ∆*fdoI* (*fdoH-sfCherry-3xFLAG*) | This study |
| pCONJ4s ∆*fdoI* (*V5-fdoH*) | This study |
| pCONJ4s ∆*fdnI* (*fdnH-sfCherry-3xFLAG*) | This study |
| pCONJ4s ∆*fdnI* (*V5-fdnH*) | This study |
| pCONJ4s ∆*cyaA* | This study |
| pCONJ4s *hybA*_G296F_ | This study |
| pCONJ4s *hybA-sfCherry-3xFLAG* | This study |
| pCONJ4s *hybA*_G296F_*-sfCherry-3xFLAG* | This study |
| pCONJ4s *hybA*_P300A_*-sfCherry-3xFLAG* | This study |
| pCONJ4s *fdoH-sfCherry-3xFLAG* | This study |
| pCONJ4s *fdoH* _P259A_*-sfCherry-3xFLAG* | This study |
| pCONJ4s *fdnH-sfCherry-3xFLAG* | This study |
| pCONJ4s *fdnH* _P259A_*-sfCherry-3xFLAG* | This study |
| pCONJ4s *hybO-sfCherry-3xFLAG* | This study |
| pCONJ4s *hyaA-T25-3xFLAG* | This study |
| pCONJ4s *hyaA-His-T18* | This study |
| pCONJ4s *V5-hybA* | This study |
| pCONJ4s *V5-hybA*_P300A_ | This study |
| pCONJ4s *V5-fdoH* | This study |
| pCONJ4s *V5-fdoH*_P259A_ | This study |
| pCONJ4s *V5-fdnH* | This study |
| pCONJ4s *V5-fdnH*_P259A_ | This study |
