# Supplementary material for: Bacterial rhomboid proteases mediate quality control of orphan membrane proteins
Source: EMBO J. 2020 Apr 27;39(10):e102922. doi: 10.15252/embj.2019102922 (PMC7232013; doi:10.15252/embj.2019102922)
Supplement: Supplementary file 6 — Table EV4 [file EMBJ-39-e102922-s006.docx]

**Table EV4. Primers used in this study**

| **Primer name** | **Sequence (5' to 3')** |
| --- | --- |
| pGL97 | AAACGCAAAAGAAAATGCCGATATCCTATTG |
| pGL98 | AATAGGATATCGGCATTTTCTTTTGCGTTTTCATTTGTAGAGCTCATCC |
| pGL154 | CATTATACCTAGGACTGAGCTAGCTGTCAATTCACTAAATAATAGTGAACGGCAGGTATATGTG |
| pGL155 | TTGACAGCTAGCTCAGTCCTAGGTATAATGCTAGCCCAACATGTTACACAATAATGGAGTAATGAACATATGAGCAAAGGAGAAGAAC |
| pGL185 | GGAAGTGTATCGTACAGTAGACGGAGTATAGTAAAACGACGGCCAGTGTTTAAACGTCATAGCTGTTTCCTG |
| pGL186 | AATAATTCCACGGACTATAGACTATACTAGCAGGAAACAGCTATGACGTTTAAACACTGGCCGTCGTTTTAC |
| pGL222 | AATTGCTAACGCAGTCAGGCACCGTGTATGCGCGGAACCCCTATTTGTT |
| pGL223 | CGCCGGACGCATCGGGCCGGATCTAGATATTTACCAATGCTTAATCAGTGAGGC |
| pGL224 | ATATCTAGATCCGGCCCGATGCG |
| pGL225 | CATACACGGTGCCTGACTGCG |
| pGL228 | CGGAGTATAGTAAAACGACGGCCAGTGTTTCGCCATACGAGTAAACGACG |
| pGL231 | CTATACTAGCAGGAAACAGCTATGACGTTTTATAGAATCAAGTAGCCTACAGGGCGGCGATTACCAGGCT |
| pGL242 | AGTCGCAAAACTCACCTTTTTTGTTGTTATCCCTCAATTACCACAAGTAAAAGGAG |
| pGL243 | CACTATACCTAGGACTGAGCTAGCCGTCAAATAACAACAAAAAAGGTGAGTTTTGCG |
| pGL244 | TTGACGGCTAGCTCAGTCCTAGGTATAGTGCTAGCCCAACATGTACAATAATAATGGAGTCATGAACATATGTTGATGATTACCTCTTTTGCTA |
| pGL245 | ATAACAACAAAAAAGGTGAGTTTTGCGACTCACCTTTTTTATTTGTTTATTTTCGTTTTCGCGCATTGAGCGAATC |
| pGL251 | GGAGTATAGTAAAACGACGGCCAGTGTTTCTTCAGAGGTAGTGCCGGAAG |
| pGL252 | GGGTAATTGAACATTTAACGGC |
| pGL253 | TCGGTTAAGCCGTTAAATGTTCAATTACCCAGGTAACATCGTGCGGATCA |
| pGL254 | ATACTAGCAGGAAACAGCTATGACGTTTCGAATCAGCCGATCGCTATAAC |
| pGL255 | TCTAGAAAGGAAGAAACACCTTATGTCTGC |
| pGL256 | GCATGCTTACGGTTCAGGGATACAAGCC |
| pGL257 | GCATGCTTAATCCATCTGCTGCTGCGC |
| pGL263 | CGGTAGCGGTAAGCTAATTGTTATTACTC |
| pGL264 | GAGTAATAACAATTAGCTTACCGCTACCGGCGTTACGCGCCGTACG |
| pGL271 | TCTAGAGGAATAAGCGACAGCAACGATGTT |
| pGL272 | GCATGCTTATTTTCGTTTTCGCGCATTGAG |
| pGL305 | CAACGCGCCACCCGCAATCAGGCCGCCAATAGCGCAAGCGTTATCTATTCCTG |
| pGL306 | ATTGGCGGCCTGATTGCGGGTGGCGCGTTG |
| pGL323 | CTATACTAGCAGGAAACAGCTATGACGTTTATGCCGCTTATCCCCAGAATGCCGAAATCA |
| pGL328 | CGGAGTATAGTAAAACGACGGCCAGTGTTTATATTAGCAAAGAATCGCGC |
| pGL353 | AAGCCCGCCAAACCACGGCCCGCTGAATTT |
| pGL354 | AAATTCAGCGGGCCGTGGTTTGGCGGGCTTGCGGGCGTGGTGTATG |
| pGL360 | TGCCCCAACGCTGATGA |
| pGL361 | AGTGAGCAGATAGTCATCAGCGTTGGGGCAGCGGGGGCGATTCTGGGA |
| pGL362 | ATACCACGACGATTTCCGG |
| pGL363 | TGTAGAAACTGCCGGAAATCGTCGTGGTATTCACTCCAGAGCGATGAAAACGTTTCAGTT |
| pGL377 | TTGCATGCTTATTCCGTAACGAGTCGGT |
| pGL378 | CTCGTTACGGAATAAGCATGCAAGCTTGGC |
| pGL414 | TATCCATATGATGTTCCAGATTATGCTTATCCCTACGATGTACCAGATTATGCATATCCATACGATGTGCCCGATTATGCATCTGCATCTTCGGTTAAGCCG |
| pGL415 | ATAAGCATAATCTGGAACATCATATGGATACATAAGGTGTTTCTTCCTTT |
| pGL416 | CGGAGTATAGTAAAACGACGGCCAGTGTTTAAGGGAAAAAATAAATATCGGCG |
| pGL417 | AATCTGTAGTTTTTCGTTAGCG |
| pGL418 | CATTTTGACGCTAACGAAAAACTACAGATTGTTGGGCATGGGGAACTTGTTTCTATTGAG |
| pGL419 | CTATACTAGCAGGAAACAGCTATGACGTTTGCAAGTAAAATGCTTGAAATAACAAGATCA |
| pGL430 | CCAGAATCAATGGCCATTAAAG |
| pGL431 | CAAGCGCAACAAATAATACAAG |
| pGL446 | CTTGCGCAGCAGCAGATGGATTAAGCATGCAAGCTTGGC |
| pGL447 | ATCCGTGGACCGGCGTGGGAATTGGCGCTGCAATCGGTGTAGTGCTCGGCGTTCTGCTGTCGTCTAGAATGAGCGATAAAATTATTCACCTG |
| pGL448 | TCCCACGCCGGTCCACGGATTTTCACGCACATACTCATCAGCACGCGCCGCCGCGACACGGGTCGGTACCTTGTCATCGTCATCCC |
| pGL449 | GAGTATCGCCTGGATCGCGTTACTGGTGGCGGTTCTTGTGACTACGTTTAAGAGCCTGTCTAGAATGAGCGATAAAATTATTCACCTG |
| pGL450 | ACGCGATCCAGGCGATACTCAGTATGGGATGACGGCCAACAAATTGCATAATTTCTTGCGGTACCTTGTCATCGTCATCCCCGAGACC |
| pGL451 | CATCATGCAATGGTTTATGTATGGCGGTGGCGTGCTGGGGCTTGGCTTGCTGCTCGGTCTGGTACTGCCGCACCTGATCTCTAGAATGAGCGATAAAATTATTCACCTG |
| pGL452 | TACATAAACCATTGCATGATGATGGTGCGCTGTTTGTCATCCAGCTGTACGCTGGCGGCATCGACCTTTTTCTGCGCCGGTACCTTGTCATCGTCATCCCCGAGACC |
| pGL453 | ATCCGCACGGTATGCCGGGGCTGTTTTACTGGTTATTGATGATCCCCGCGCATCTGTTTATTTTTCGCGGATCTAGAATGAGCGATAAAATTATTCACCTG |
| pGL454 | CCCCGGCATACCGTGCGGATGCCAGAAAGCGCGGACATCGATAGTACGATAGTCGCCTTTATCTTCCAGGCTCGGTACCTTGTCATCGTCATCCC |
| pGL458 | TCAGGTGAATAATTTTATCGCTCATTCTAGAAATCGTTTTCCTCAACATGTAGTAGACCACAACGCCCGCAACCACAAGTCCACCGCCAAAACGACGTAAAATATTATCGGGC |
| pGL459 | CGGGTCTCGGGGATGACGATGACAAGGTACCGACTCCGCGTTCACAGAAACCGGATGTTAACGCTAAAGAGGGCGGCAACGTCTCTGCAGGCGCTATTGGTT |
| pGL460 | TCAGGTGAATAATTTTATCGCTCATTCTAGACACCGCCATCACGCTGACACCGGCAACCAGCCCAACCACACCGCCGAGCAAACCAATAGCGCCTGCAGAGA |
| pGL461 | GGTCTCGGGGATGACGATGACAAGGTACCGAATAACCACGCTACTGTGCAATCTTCCACGCCGGTTTCTGCTGCGCCACTGCTGCAGGTCAGCGGCG |
| pGL462 | CAGGTGAATAATTTTATCGCTCATTCTAGATACCAGCCAGGCAGCAGCGAGGATCAGGGCAATAATGGCGATGAGTGCGCCGCTGACCTGCAGCAGT |
| pGL463 | GGTCTCGGGGATGACGATGACAAGGTACCGTTGACCAAGAACGTCAAGGTTGTCGGTGAACCGCCTGAAGAACCAAGCCTGCTGGCTTCTATCTTCATCTCCTGGTTCCC |
| pGL464 | CAGGTGAATAATTTTATCGCTCATTCTAGAGCCGCCCTGCATTTGACGCATGAAGAAGATCCACACACCAATCAGCAACAGCATCGGGAACCAGGAGATGAAGATAGAAG |
| pGL465 | GGTCTCGGGGATGACGATGACAAGGTACCGCAACAGGTGACGTTTAATTATCTGTTAGCGCAAGGGGAGTACCGTATTTCCACATTGCTGGCGGTATTGTTTGC |
| pGL466 | CAGGTGAATAATTTTATCGCTCATTCTAGAAACTCGCAGCCAGAACAGGCCACAAATCAACCAACCGATAGCAACCCCCGCAGCAAACAATACCGCCAGCAATG |
| pGL467 | GGTCTCGGGGATGACGATGACAAGGTACCGCAGTATTGGGGAAAAATCATTGGCGTGGCCGTGGCCTTACTGATGGGCGGCGGCTTT |
| pGL468 | CAGGTGAATAATTTTATCGCTCATTCTAGACATATGGCCAATTAACAGGCCTAACACTACGCCCCAAAAGCCGCCGCCCATCAGTA |
| pGL469 | GGTCTCGGGGATGACGATGACAAGGTACCGTATGTTTATACAAATGTACAACCTGTTAAGGAAATTAAATCGGAATTTTTAACAAATGAAGTAATATATTTATTTCTCGGG |
| pGL470 | CAGGTGAATAATTTTATCGCTCATTCTAGAGAACCACCCTGTTTTGAATGCCCATACCAAAAGAATGACAACTAGTACAGCCATCCCGAGAAATAAATATATTACTTCATTTG |
| pGL471 | GATGACGATGACAAGGTACCGGGCGACGTTTATTACGTAGGTCATCTGCCGTGGTTCGAGCGCTTGTGGTATGCGCTGGCAAACCATCCGATTCTGCT |
| pGL472 | CAGGTGAATAATTTTATCGCTCATTCTAGACAGCAGACGCCACAGTACCCATGCCAGCAATATCACACTGATTGCCGCCAGCACCGCCAGCAGAATCGGATGGTTTGCC |
| pGL473 | GGTCTCGGGGATGACGATGACAAGGTACCGAACGAAGTTGTAAATTCAGGCGTGATGAACATTGCGTCTTTGGTTGTATCGGTGGTGG |
| pGL474 | CAGGTGAATAATTTTATCGCTCATTCTAGAGATAAAAAACCACAAGATGAGCCCGATAAGAAGAACCACCACCGATACAACCAAAGAC |
| pGL475 | GGGTCTCGGGGATGACGATGACAAGGTACCGTATTACTATCGGGCGAAGCAGGCAGTTTATCGTGCTGATGACTACGTCCACGAAAAACCCTGGCAAGGA |
| pGL476 | CAGGTGAATAATTTTATCGCTCATTCTAGATGCCAGCAACAGTCCTAGTACCAGCCCAACGGCCGCGCCCACACCAATTCCTTGCCAGGGTTTTTCGT |
| pGL477 | GGTCTCGGGGATGACGATGACAAGGTACCGCGTAAACATCCGGGGCTGTTTATCGGTATGTACGTTGCTTTTTTTGCCACCCTGGCGGTGATGTTGCAGTCC |
| pGL478 | CAGGTGAATAATTTTATCGCTCATTCTAGAAAGCAGGATAAATAATACAACCAGTAGCCAGACAGAGCCTGACAGCGTTTCGGACTGCAACATCACCGCCA |
| pGL479 | GGGATGACGATGACAAGGTACCGGCCGCACGCGATGCCGTTGGCTGCGCGGATTCTTTTGTTCGTGAAAGACCCTGGTGTAGCGTGGGTA |
| pGL480 | CGTCAGTCAGGTGAATAATTTTATCGCTCATTCTAGAGCGCATGCTTAACAGTGCGCCGATAAAAATACCTACCGCAGCTGCTGTACCCACGCTACACCAGGGT |
| pGL481 | CGGGTCTCGGGGATGACGATGACAAGGTACCGTCAAATATCACCTTTTCTCATGCTTTATTCTATCAAAAATCGCTCATTTTTACTTTTTTTACCATAATGTTACTCAG |
| pGL482 | CAGGTGAATAATTTTATCGCTCATTCTAGACGAGATATAAAAAACAACGTGTAAGTTATGTAAATTGCCGATGCTACTGAGTAACATTATGGTAAAAAAAG |
| pGL483 | GGTCTCGGGGATGACGATGACAAGGTACCGGTGTGTGCGATGAAAGCCACGGCAGAAGGGCCGAAGCGGATGGATTGTGCATCTGGATTTGGCGCGGCA |
| pGL484 | CAGGTGAATAATTTTATCGCTCATTCTAGACAGCGCATGAGAAACCGCAACAAACCCAAAGGTGGCGGTCACCATCGTTGCCGCGCCAAATCCAGATG |
| pGL485 | GGGATGACGATGACAAGGTACCGAATGCGGCACTGGAATCTTGCCGGGACTTACCGCAGGGAAGCCGACCGCTGCTGGTGAGCCATGGTATT |
| pGL486 | CAGGTGAATAATTTTATCGCTCATTCTAGATGCCCATGCTGGTAATCCGAGAATCGTACTCACCAGACATCCCAGTGCAATACCATGGCTCACCAGCAG |
| pGL487 | GGTCTCGGGGATGACGATGACAAGGTACCGTTTTTTATTTCTGATGCGGTAGCGGCAACGGGTGCACCGGCGCAAGGTAGCCCGATGTCTTTGATTT |
| pGL488 | CAGGTGAATAATTTTATCGCTCATTCTAGAACGCAGGATCATGAAATAGAAAATCAGACCGAATACCACCAGCATCAAAATCAAAGACATCGGGCTACC |
| pGL489 | GGGATGACGATGACAAGGTACCGAATGCTATCTGGATTGCCGTTGCCGCCGTGAGCCTGCTGGGCCTGGCG |
| pGL490 | CAGTCAGGTGAATAATTTTATCGCTCATTCTAGAGGAGGCATAACCCAGAATGGCGCCAAACGCCAGGCCCAGCAGGCTCA |
| pGL491 | GGTCTCGGGGATGACGATGACAAGGTACCGTACGTCCATGACTTACCGGGCGGCATTCGTGTCTCGTTATCCATTGATATTCTTTACTTTATCACGTCTTCGTGGAA |
| pGL492 | CAGGTGAATAATTTTATCGCTCATTCTAGACATTCGGACCATATTCAGCAAAATTAACGCCGTCAAAATCCAGAACAGAACGCTTTTCCACGAAGACGTGATAAAGT |
| pGL493 | GATGACGATGACAAGGTACCGTCAGCTTCCCTGAAGAATCAACAAGGCTTTAGCCTGCCGGAGGTAATGGTTGCGATGGTG |
| pGL494 | CAGGTGAATAATTTTATCGCTCATTCTAGACTGGATACCCGATAACGCAGTGACAATCATCACCATCAGCACCATCGCAACCATTACCT |
| pGL495 | GGATGACGATGACAAGGTACCGAGCCGCGTGGTCGATATTCCGCAAATGGGTACTCATTCCACCGCCGATACCGTCGGTTTAACCGCGCTT |
| pGL496 | CAGGTGAATAATTTTATCGCTCATTCTAGAAACGGCGCTGGCGACTGCGTGCACACCAACAGCCGCAGCCACCACACCAAGCGCGGTTAAACCGACGG |
| pGL497 | GGTCTCGGGGATGACGATGACAAGGTACCGACGACCACTACCGGCGCAACCACGACTGCTACTACCACTGGTGGCGTGGCTGCTGGCGCTGTCGGCACGGCGA |
| pGL498 | CAGGTGAATAATTTTATCGCTCATTCTAGATGCAACAACTGCCAACGTCGCCACACCAACAGCCGTTGCCACACCGACAACTGTCGCCGTGCCGACAGCGCCA |
| pGL499 | GGATGACGATGACAAGGTACCGGGAGCCTCCTTCGGAGCAGGCGCTTCCGCTACGCTGTTTGGTTCAAGTGGTTCTGGTAACTTCATGACCCGCATG |
| pGL500 | CAGGTGAATAATTTTATCGCTCATTCTAGAGATGTTACCCAGCACCAGACTGATGATAAAGAATAACGTTGCCAGCAGCGCCGTCATGCGGGTCATGAAGTTAC |
| pGL501 | GGGATGACGATGACAAGGTACCGGAAACCGTCGAGCCGGAGCCGCAACCGCAGCCAATGCCCGTTGACGATGGCGGGCTGGGCTGTCTGGGCGTCATCAAA |
| pGL502 | CAGGTGAATAATTTTATCGCTCATTCTAGAGAAGAGATGGAGTATTTTTCCTATCAAACCGGCAAAGATAAAAATGTAGAAAATAATTTTGATGACGCCCAGACAGC |
| pGL508 | GGTCTCGGGGATGACGATGACAAGGTACCGGACACCCATACGCTCGCCACCGAAGCCCAACAGGTGGATAACACCACTAACATTTTACGCCTGATGTTG |
| pGL509 | TCAGGTGAATAATTTTATCGCTCATTCTAGATAGCAGCGTGCGGGTCAGCACTACGCCGATTGCCAGTGAGAGCAGCAACATCAGGCGTAAAATGTTAG |
| pGL516 | CGGGTCTCGGGGATGACGATGACAAGGTACCGGATGATATTGTTAATTCTGTGCCCTCCTGGATGTTTACCGCGATTATTGCCGTATGCA |
| pGL517 | CAGGTGAATAATTTTATCGCTCATTCTAGAATAGAGCCTGGCGAAAATAATTCCAATAATAAACAGAATGCATACGGCAATAATCGCG |
| pGL518 | GGTCTCGGGGATGACGATGACAAGGTACCGAATCCTTTTCCACCGATGATGGACACGTTGCAAAATATGGCAACGCGTCCCGCGCTGTGGA |
| pGL519 | CAGGTGAATAATTTTATCGCTCATTCTAGAAACCAGCCAGACCAGCATCAGGATAATCGCGACTAACAGTATCCACAGCGCGGGACGCGTT |
| pGL520 | GGATGACGATGACAAGGTACCGACCGAACGCTACGGAGATTTTGTTCGCTATAACCCACCGTTAACGGGTCAGACGCTGGTGTTGTGGGCG |
| pGL521 | CAGGTGAATAATTTTATCGCTCATTCTAGACACTCGCCAGAGGATCAGTGCCATCAGCAATAACAACACCACTGGCAGCGCCCACAACACCAGCGTCT |
| pGL522 | CGGGTCTCGGGGATGACGATGACAAGGTACCGACCTGGGAATATGCGCTAATTGGGTTAGTCGTCGGCATCATTATTG |
| pGL523 | CAGGTGAATAATTTTATCGCTCATTCTAGAATTACCAAAACGCATGGCCACAGCACCAATAATGATGCCGACGACT |
| pGL524 | TCTCGGGGATGACGATGACAAGGTACCGACCCCTGCATTTGCTTCCTGGAATGAATTTTTCGCAATGGGCGGTTACGCCTTTTTTGTCTGGCTGGCGG |
| pGL525 | CAGGTGAATAATTTTATCGCTCATTCTAGACATCACCGAGTGCACGACCAAAACCACCAGCGGAATAACGGTCATCACCACCGCCAGCCAGACAAAAAAG |
| pGL526 | CGGGTCTCGGGGATGACGATGACAAGGTACCGATGCAGGATTTGCGTCTGATATTAATCATTGTTGGCGCGATCGCCATAATC |
| pGL527 | CAGGTGAATAATTTTATCGCTCATTCTAGAGCTGGTCCAGAAACCATGTACCAGTAAAGCGATTATGGCGATCGCGCCAAC |
| pGL528 | GGATGACGATGACAAGGTACCGAGCAAACCACCTCTTTTCTTTATTGTTATCATTGGCTTAATTGTCGTC |
| pGL529 | CAGGTGAATAATTTTATCGCTCATTCTAGACATAAAACGAAACGATGCGGCGACGACAATTAAGCCAATGATAAC |
| pGL530 | GGGCGGGTCTCGGGGATGACGATGACAAGGTACCGTCTGCCCAGTTCTGGAATCGCGCTTCCATAAAACAAAAAATCCCTCTCACCATCCTCTACATTC |
| pGL531 | CAGGTGAATAATTTTATCGCTCATTCTAGATGTGTGACGGAAGAATAAACGTAGAAAAACGCTAAACAAGAGAATGTAGAGGATGGTGAGAGG |
| pGL538 | GGTCTCGGGGATGACGATGACAAGGTACCGAAACTGGACGATCTTTCTACCGGTGCGCGTTCCGAAAATATTCAACACACCCTGTATAAAGGCATGA |
| pGL539 | CAGTCAGGTGAATAATTTTATCGCTCATTCTAGAGCGACGAACCAGCACGGTTAAGCCCGCCAGCACAGCCAGTGGTAGCATCATGCCTTTATACAGGGTGTG |
| pGL540 | GGTCTCGGGGATGACGATGACAAGGTACCGGTACGTCGCGACCTGAATATGGAAGCGGTTGTCGCACCAGAGATTTCTGCGACGGTGGGCGTGGCTGCGGCTT |
| pGL541 | CAGGTGAATAATTTTATCGCTCATTCTAGACAGCACTTTACTAGCGGCAAAAACTGCCGCGCCAACAATGGGGTTAACCGCAAAAGCCGCAGCCACGCCCACC |
| pGL542 | GGATGACGATGACAAGGTACCGGGCACCGGATCTGGCGGTGGCAGTTCATCGCAAGGCCCGCGCCCGCAGCTTGGCGGTCGTGTCGTTACCATC |
| pGL543 | CAGGTGAATAATTTTATCGCTCATTCTAGAAATGGTATAGAAACCACTGGCCGCCCAGATAATGACAATCGCTGCCGCTGCGATGGTAACGACACGACCGC |
| pGL544 | GGTCTCGGGGATGACGATGACAAGGTACCGATGAATATTCTCCACTTCTCACAGTCGGTAAAATGGAGCAGCTGGTTTATCTGTTCTCTACTGC |
| pGL545 | CAGGTGAATAATTTTATCGCTCATTCTAGAGGAAAAACGCCAGATAAACGCAAGGAAAATCAGTCCATGAAGCAGTAGAGAACAGATAAACCAGC |
| pGL546 | GGTCTCGGGGATGACGATGACAAGGTACCGGAACAGTACAATATTCCGCTGTCTGAGTGGACGACATCAATGTATGTGATTCAGTCATCGATGATTTTTGTTTATAGCC |
| pGL547 | AGTCAGGTGAATAATTTTATCGCTCATTCTAGAGCCGCCAAGGAAATAAATTCCCAACGGGATTGCCAGCAACACAGTAAAGACCAGGCTATAAACAAAAATCATCGATGACTG |
| pGL548 | GGTCTCGGGGATGACGATGACAAGGTACCGGAATTGTTGTCTGAATATGGTTTGTTTTTGGCGAAAATCGTTACCGTTGTGCTAGCG |
| pGL549 | CAGGTGAATAATTTTATCGCTCATTCTAGAAGCAACATTGACAATAATGGCGGCAATCGCCGCAATCGCTAGCACAACGGTAACGA |
| pGL550 | GGTCTCGGGGATGACGATGACAAGGTACCGAATCAGCACGGACTGGCGCATCAGGAAAAAGCCAGTGCACGCGGGCAATACAGCCTGTTATTGCTGGGG |
| pGL551 | CAGGTGAATAATTTTATCGCTCATTCTAGAATAAACCACGCGCCAGAGGATCAGAATCAGTGCGCAAAGTGAAACCATCCCCAGCAATAACAGGCTGT |
| pGL552 | GGTCTCGGGGATGACGATGACAAGGTACCGCTGCCGAAAGATCCGAAGATCGACACCTCGGTAAGCCTGTGGAAAGGCGCGTTGAAACCGCTGGCAGCGGCT |
| pGL553 | CAGGTGAATAATTTTATCGCTCATTCTAGAGCCAATACCGATGTAGTGGAAAATCAACCCGGCAAAAGTGGCAATAAAGCCAGCCGCTGCCAGCGGTTTCA |
| pGL554 | GGGATGACGATGACAAGGTACCGTTGCCGGAGAACCCGGAAATCAGCGAAACCGTGAAATTCTGGAAAGGCATCTGGAAACCGCTCGCGGCTGTTG |
| pGL555 | CAGGTGAATAATTTTATCGCTCATTCTAGACGGACCGACACCCACGTAGTGGAAGATACTGGCCGCAAAGGTAGCCGCAAAGCCAACAGCCGCGAGCGGTTTC |
| pGL564 | CGGAGTATAGTAAAACGACGGCCAGTGTTTCATCTCACGTGAAGCGATGG |
| pGL565 | ATAAGACTGAAAGCGATACTTACCGTCTTTCAGCGGCGGATTACCTTCT |
| pGL566 | AAAGACGGTAAGTATCGCTTTCAG |
| pGL567 | CTATACTAGCAGGAAACAGCTATGACGTTTATCCACAATGCAGAGAGATTTG |
| pGL568 | GCGCCAGTGCAGAAGGATGAGC |
| pGL569 | CGATATCAAAATGCGCACCGG |
| pGL570 | CGGAGTATAGTAAAACGACGGCCAGTGTTTGATATATAGCGAATGAGGTGAACGAG |
| pGL571 | TTATAGTTATTCCGTTGCGAAGACC |
| pGL572 | TGCCAGGTCTTCGCAACGGAATAACTATAATGATTAGCTTTCGTAGGCCTG |
| pGL573 | CTATACTAGCAGGAAACAGCTATGACGTTTTTTTACCATTACGCGCCGATG |
| pGL574 | CTGGCTGTTCTGGTTACAGGG |
| pGL575 | GCGCAAAAGTCGTTCTTTGCC |
| pGL584 | cggagtataGTAAAACGACGGCCAGTGTTTATGCAGCAGGAAGGCATGG |
| pGL587 | ctatactagCAGGAAACAGCTATGACGTTTTTTTCATCGACTCTTTCGTACCG |
| pGL612 | CGGAGTATAGTAAAACGACGGCCAGTGTTTGGATCACATCCGCAAAGCG |
| pGL615 | CTATACTAGCAGGAAACAGCTATGACGTTTACTTCGGCACGTTGTACGG |
| pGL617 | CTGGTTGCACAGCTCGCAC |
| pGL618 | CCATCCCATTAAAAGATAACGG |
| pGL619 | CGGAGTATAGTAAAACGACGGCCAGTGTTTGTCAACAAAGACCAGGAGGAG |
| pGL620 | CAGCCAGTGGTAGCATCATAAATTTATACAGGGTGTGTTGAATATTTTCG |
| pGL621 | TGTATAAATTTATGATGCTACCACTGGCTGTG |
| pGL622 | CTATACTAGCAGGAAACAGCTATGACGTTTAACATAACCTTGTTCAGACGC |
| pGL623 | CAGCCAGTGGTAGCATCATAAA |
| pGL624 | CCGAGATCATCAGTGACCCC |
| pGL625 | CCTGGTCGAACAACGACAAAC |
| pGL626 | TTCTTTGATGATGGCCATGTTATCTTCTTCTTCTCCCCGTGAGTCAGCG |
| pGL627 | GAAGAAGATAACATGGCCATCATC |
| pGL628 | TCATCCTTGTAATCGATATCATGATCTTTATAATCACCGTCATGGTCTTTGTAGTCACCACCGGTACTATGACG |
| pGL629 | TAAAGATCATGATATCGATTACAAGGATGACGATGACAAGTAAACGGGGAGAATAACCGTG |
| pGL630 | TTCTTTGATGATGGCCATGTTATCTTCTTCTGACTCATGATCGTCTCCTCC |
| pGL631 | TAAAGATCATGATATCGATTACAAGGATGACGATGACAAGTAAACGGAGGAGACGATCATGAG |
| pGL632 | TTCTTTGATGATGGCCATGTTATCTTCTTCTGACTCATGATCGTCTCCTCCGTCGTGATGGTCGTTTTTGGTGTTGCGACGAACCAGCACGG |
| pGL633 | TAAAGATCATGATATCGATTACAAGGATGACGATGACAAGTAATAAAAGCTTGGCACTGGCCG |
| pGL640 | CGAGCACTTCACCAACAAGGACCATAGCATATGACTGGAGATAACACCCTC |
| pGL641 | TTCTTTGATGATGGCCATGTTATCTTCTTCTTCTCCCCGTGAGTCAGCG |
| pGL653 | CGGAGTATAGTAAAACGACGGCCAGTGTTTTTGGCTGCGGTTATTGCATTG |
| pGL715 | CACTAAATAATAGTGAACGGCAGG |
| pGL716 | CACTGATGAGAATATCGTCGG |
| pGL717 | CATACACCACGCCAGAAAGC |
| pGL718 | CAGATCTTTGGTTTCCATGCTGC |
| pGL726 | TGTATAAAGGCATGATGCTAGCT |
| pGL739 | CCCTGTATAAAGGCATGATGCTAGCTCTGGCTGTGCTGGCGGGC |
| pGL740 | TAGCATCATGCCTTTATACAGGG |
| pGL741 | GCTGCTGGGTCTGGATAGCACCAACCGCCCGCCAATTCC |
| pGL742 | TGCTATCCAGACCCAGCAGCGGATTCGGAATCGGTTTACCTTCAGCAGCCGCATGACTG |
| pGL746 | CGGAGTATAGTAAAACGACGGCCAGTGTTTATTTCTGGTCTGGATACCGG |
| pGL747 | GCTCAAACTCTCTTTAATCACGCC |
| pGL748 | GCTGGCGGCGTGATTAAAGAGAGTTTGAGCGAGCGTTACAGCATTGAGCG |
| pGL749 | CTATACTAGCAGGAAACAGCTATGACGTTTTAATAAATTGCTCATCAGATGGCCTC |
| pGL751 | GCTGGCGATGGATTCATCC |
| pGL752 | GCCGCAACTGGGTGATTTAATC |
| pGL767 | ctatactagCAGGAAACAGCTATGACGTTTAATCAGGTTCACTTCCGGCG |
| pGL809 | ATCCTCTAGAGTCGACCTGCAGGCATGCATTATTTTCGTTTTCGCGCATTG |
| pGL810 | CAGGAAACAGCTATGACCATGATTACGCCAGGAATAAGCGACAGCAACGATG |
| pGL845 | CGGTTTACTGATGATTTTGCCG |
| pGL846 | CGGCAAAATCATCAGTAAACCGCGTCATGAGGCGAGCAAAG |
| pGL880 | TAAAGATCATGATATCGATTACAAGGATGACGATGACAAGTAAGGTACCGTGAGGTCCGC |
| pGL881 | CTATACTAGCAGGAAACAGCTATGACGTTTCTCCTGAGTCGATGCCTTC |
| pGL882 | CGGAGTATAGTAAAACGACGGCCAGTGTTTATTTCCAGTCGGAAAACTGC |
| pGL883 | TTCTTTGATGATGGCCATGTTATCTTCTTCCTCATGATGATCCTCCTCGTCATC |
| pGL884 | TAAAGATCATGATATCGATTACAAGGATGACGATGACAAGTAACGATGACGAGGAGGATCATC |
| pGL885 | CTATACTAGCAGGAAACAGCTATGACGTTTAGCGAACAACCTGCATCG |
| pGL886 | TAAAGATCATGATATCGATTACAAGGATGACGATGACAAGTAATCTCTTTTGAACTTTAAGCTGAAAATGGC |
| pGL887 | CTATACTAGCAGGAAACAGCTATGACGTTTATTATTCAGGAATCACTGGACG |
| pGL888 | GGCGCTTTATCGCTGCTG |
| pGL889 | CTGTGAAGATCCCGGCTG |
| pGL890 | CCCAGAATGCCATATACATATGG |
| pGL891 | GAAGGAACGCTACTGAGAG |
| pGL894 | GTTTAAACGAATTCGAGCTCG |
| pGL897 | CCCGGGTACCGAGCTCGAATTCGTTTAAACCGGAATTGCCAGCTGG |
| pGL749 | CTATACTAGCAGGAAACAGCTATGACGTTTTAATAAATTGCTCATCAGATGGCCTC |
| pGL751 | GCTGGCGATGGATTCATCC |
| pGL752 | GCCGCAACTGGGTGATTTAATC |
| pGL904 | CTATACTAGCAGGAAACAGCTATGACGTTTTAATAAATTGCTCATCAGATGGCCTC |
| pGL905 | CGGAGTATAGTAAAACGACGGCCAGTGTTTATCATTACTTACATGGTGACCTTTG |
| pGL906 | TTCTTTGATGATGGCCATGTTATCTTCTTCTGCCTGTTTATCCTCATTGCC |
| pGL907 | TAAAGATCATGATATCGATTACAAGGATGACGATGACAAGTAATGAGGATAAACAGGCATGAGC |
| pGL908 | CTATACTAGCAGGAAACAGCTATGACGTTTAAGAAATAGCCAGGGGATG |
| pGL909 | CACCGACAAACCCATTATC |
| pGL927 | GGGGTCGAATGGAAAGCCAGTACCGATTTCTGACTCATGATCGTCTCCTCC |
| pGL928 | GAAATCGGTACTGGCTTTCC |
| pGL929 | TCATCCTTGTAATCGATATCATGATCTTTATAATCACCGTCATGGTCTTTGTAGTCACCGGAAATCTCCAGAGTAG |
| pGL963 | CTGGAAAGCTCTCGCGGCTGTTGGCTTTG |
| pGL964 | GCAAAGCCAACAGCCGCGAGAGCTTTCCAGATGCCTTTCCAGAATTTCACG |
| pGL965 | CTATACTAGCAGGAAACAGCTATGACGTTTTTTTCTGCATAACCGGACC |
| pGL966 | GAAAGGCATCTGGAAAGCT |
| pGL970 | CCAGAGCTTTCAACGCGCCTTTCC |
| pGL971 | TGTGGAAAGGCGCGTTGAAAGCTCTGGCAGCGGCTGGCTTT |
| pGL972 | GAAAGGCGCGTTGAAAGCT |
| pGL1060 | TCATTTGCGCTCCTCGTC |
| pGL1061 | CACGAAGAGAAAGACGAGGAGCGCAAATGAGGTACCGTGAGGTCCGC |
| pGL1101 | cggagtataGTAAAACGACGGCCAGTGTTTTCTTTTACAAATAAGCCTTTTCCGC |
| pGL1102 | TATCGCCTGATTTGCTACCC |
| pGL1103 | CAGTCATGACGGGTAGCAAATCAGGCGATATATTACAGCAATATTTTTCGTGATG |
| pGL1104 | ctatactagCAGGAAACAGCTATGACGTTTAATGAAGAGACAAATCTTTCGC |
| pGL1105 | CTTTCATTGAATGTACGGCG |
| pGL1106 | GCTCGGTAAACCTGATCATAATG |
| pGL1125 | ACTCATGATCGTCTCCTCCG |
| pGL1126 | CATCACGACGGAGGAGACGATCATGAGTCAatgcagcaatcgcatcagg |
| pGL1127 | TCATCCTTGTAATCGATATCATGATCTTTATAATCACCGTCATGGTCTTTGTAGTCccgccgcgtgcgcg |
| pGL1132 | TCATGACGATTATGATCGTTTTGTTTTAAAG |
| pGL1133 | TTTAAAACAAAACGATCATAATCGTCATGAGGCGAGCAAAGCAcatcaccatcaccAtcacagcgctgccgccagcgaggc |
| pGL1134 | AATTCTCTGGCTCATGCTTTGCTCGCCTCATGACGTTAgcgttccactgcgcccag |
| pGL1135 | GAGCAAAGCATGAGCCAGAGAATTACTATTGATCCGGTAACCCG |
| pGL1312 | ATGACCATGATTACGAATTCCCGGATTAATACGGGGAGAATAACCGTGAAC |
| pGL1313 | TTCCGGTTTAAACGAATTCGAGCTCGGTACTCATGACTCATGATCGTCTCC |
| pGL1314 | CCTCTACAAAACAGACCTTAAAACCCTAAAGGCTTAAGAGAGAATATAAAAAGCCAGATTATTAATCCGGCTTTTTTATTATTTCAAGCGAGCTCTCGAAC |
| pGL1315 | TTTAGGGTTTTAAGGTCTGTTTTGTAGAGGAGCAAACAGCG |
| pGL1316 | ACACGAAAAACAAGTTAAGGGATGCAGTTTCATATGTCAGATCCTTCCGTATTTAGC |
| pGL1317 | AAACTGCATCCCTTAACTTG |
| pGL1376 | CTATCCAGACCCAGCAGCGGATTCGGAATCGGTTTACCCATATGTCGTCTCCGTTACAC |
| pGL1377 | GCTGCTGGGTCTGGATAGCACCGCTTATCAATCGCAAGATATCATTCG |
| pGL1378 | TTACTCATGATGATCCTCCTCG |
| pGL1379 | GACGATGACGAGGAGGATCATCATGAGTAATCTCTTTTGAACTTTAAGCTGAAAATG |
| pGL1380 | cggagtataGTAAAACGACGGCCAGTGTTTATCAACAAAATGGCGGAAGG |
| pGL1381 | CTATCCAGACCCAGCAGCGGATTCGGAATCGGTTTACCCATCTGTTCGCCCCCTTAC |
| pGL1382 | GCTGCTGGGTCTGGATAGCACCGCTATGGAAACGCAGGATATTATC |
| pGL1383 | ctatactagCAGGAAACAGCTATGACGTTTACAGGTTTTCACACAAGCC |
