# Supplementary figures and images for: Bacterial rhomboid proteases mediate quality control of orphan membrane proteins
Source: EMBO J. 2020 Apr 27;39(10):e102922. doi: 10.15252/embj.2019102922 (PMC7232013; doi:10.15252/embj.2019102922)

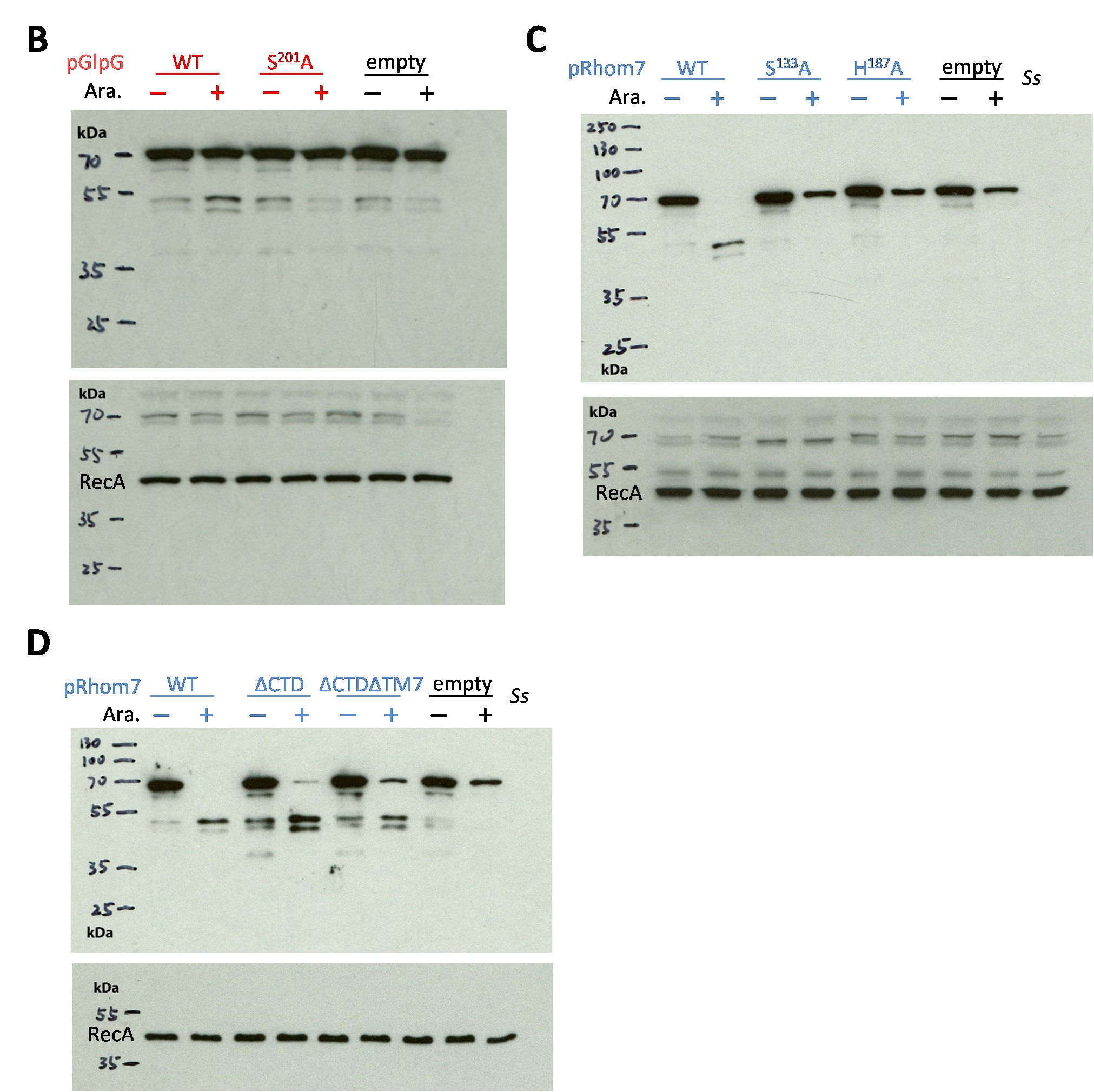

Supplement: Supplementary file 8 — Source Data for Figure 1 [file EMBJ-39-e102922-s007.jpg]

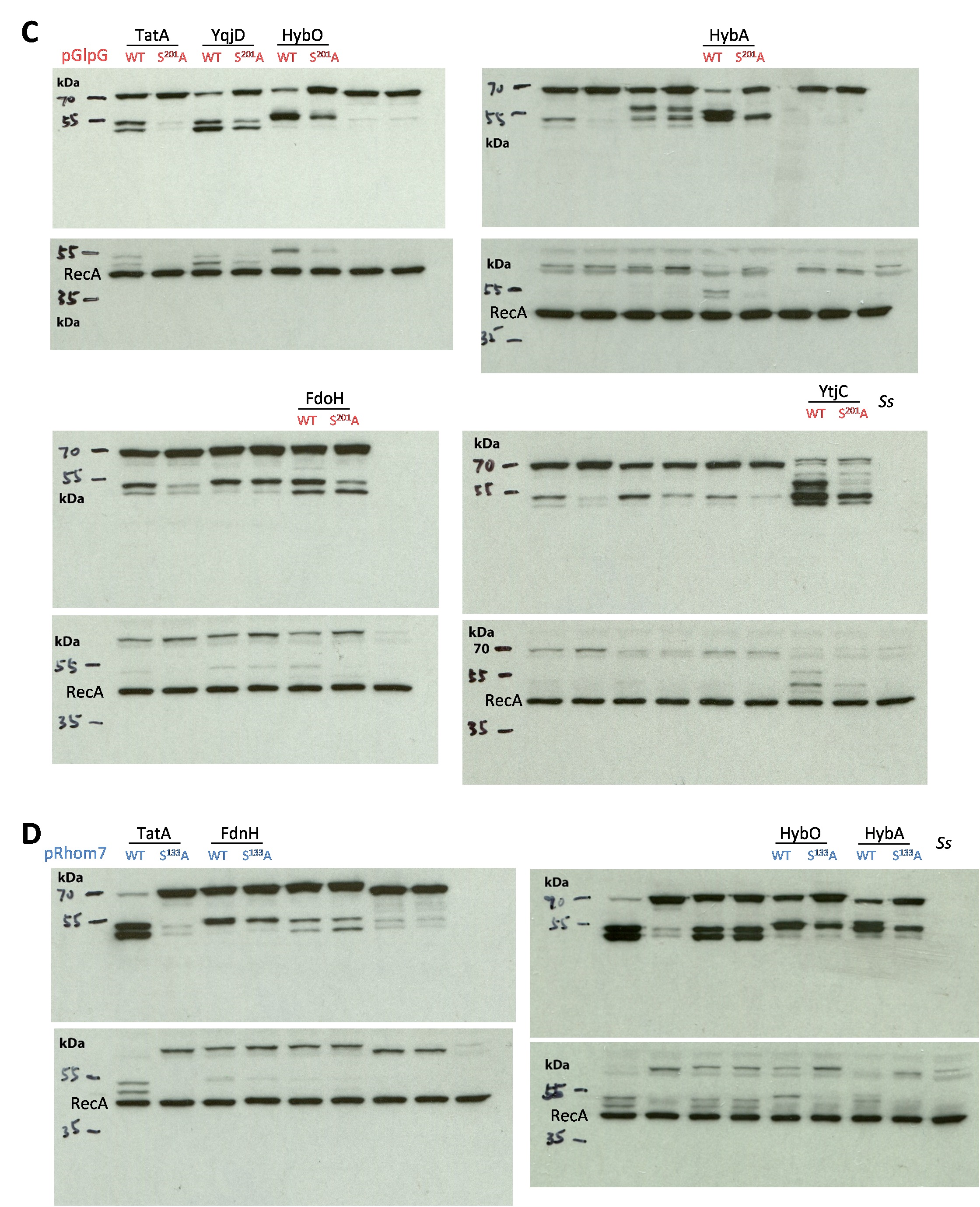

Supplement: Supplementary file 9 — Source Data for Figure 2 [file EMBJ-39-e102922-s008.jpg]

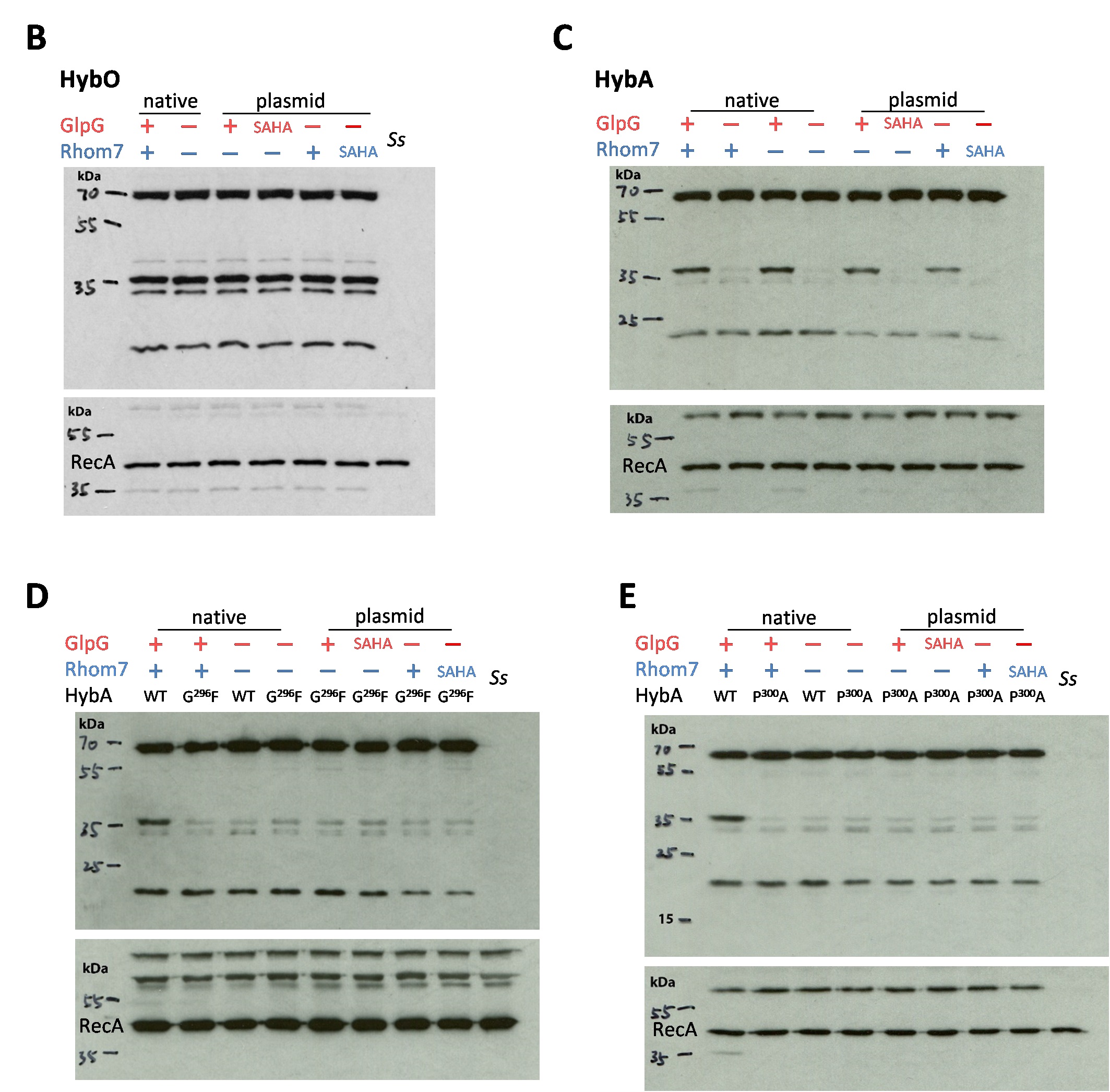

Supplement: Supplementary file 10 — Source Data for Figure 3 [file EMBJ-39-e102922-s009.jpg]

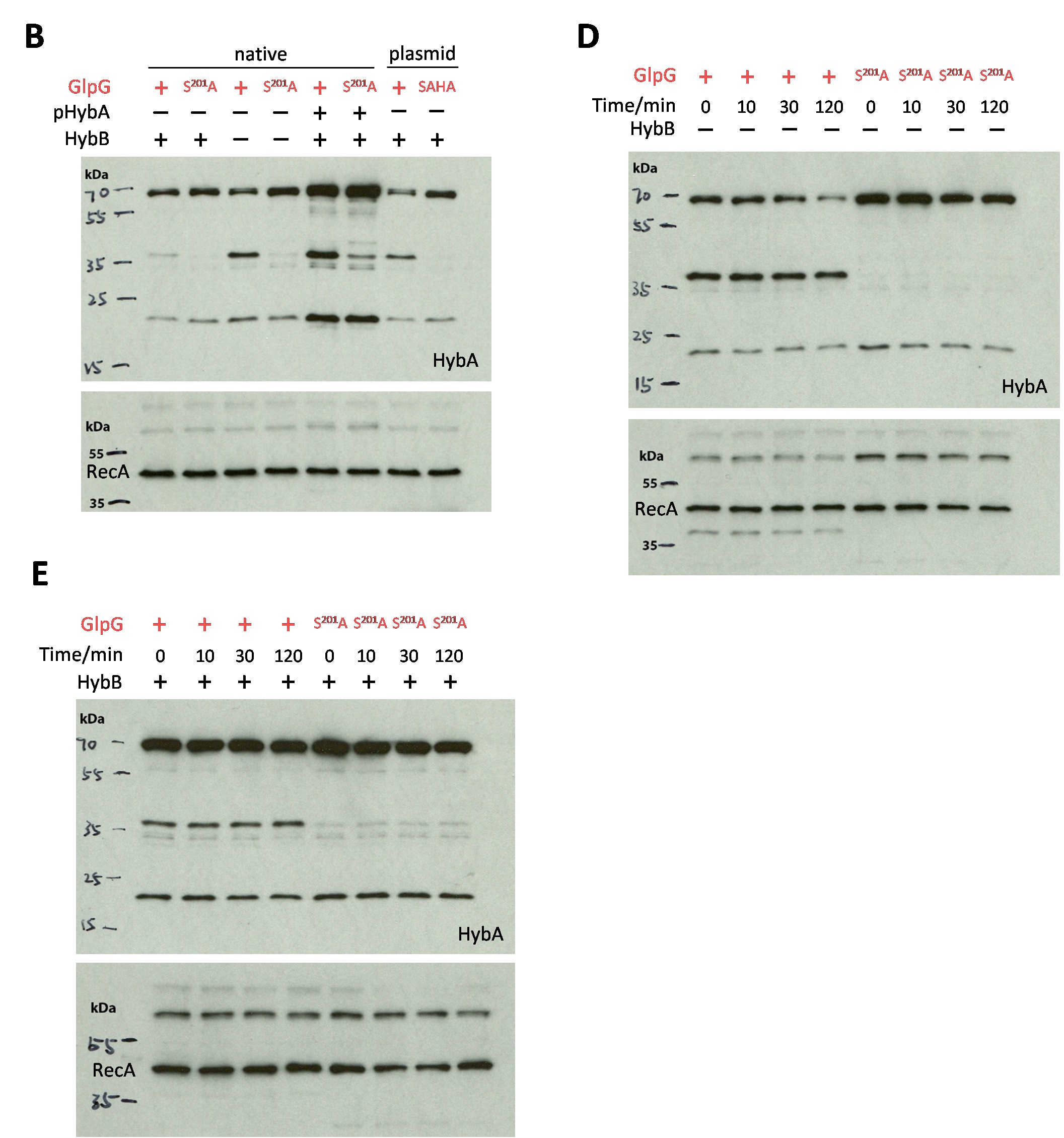

Supplement: Supplementary file 11 — Source Data for Figure 4 [file EMBJ-39-e102922-s010.jpg]

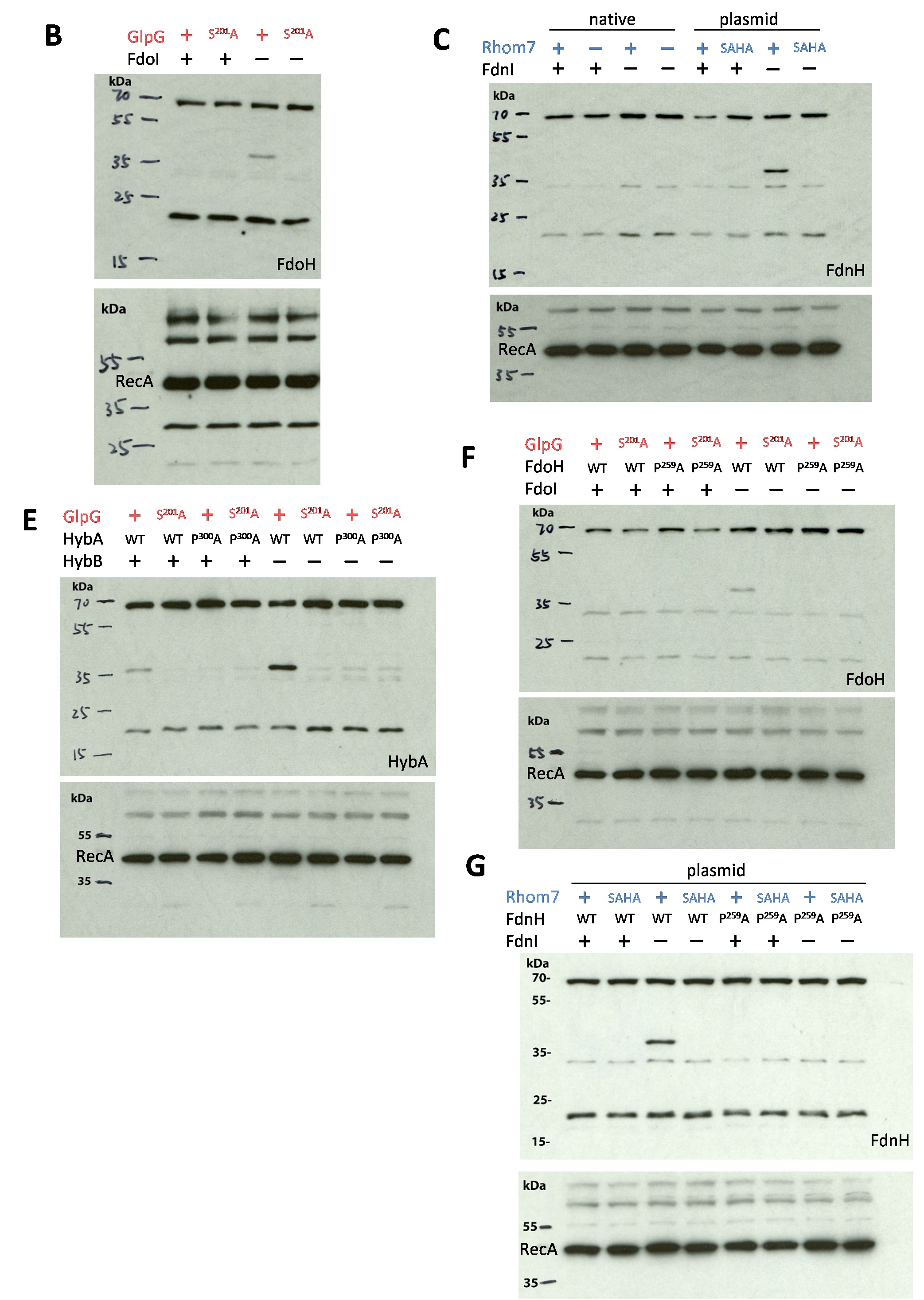

Supplement: Supplementary file 12 — Source Data for Figure 5 [file EMBJ-39-e102922-s011.jpg]

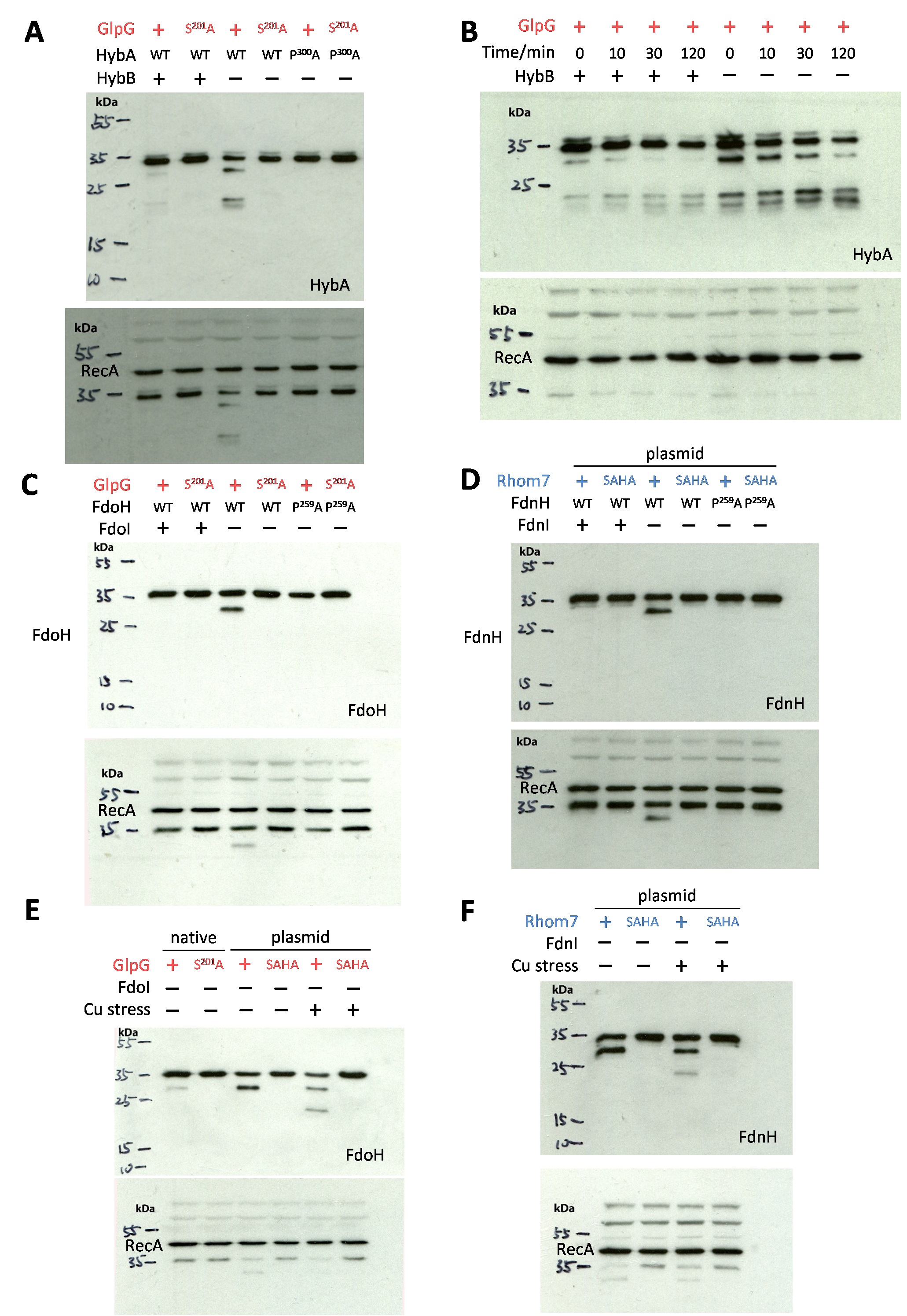

Supplement: Supplementary file 13 — Source Data for Figure 6 [file EMBJ-39-e102922-s012.jpg]

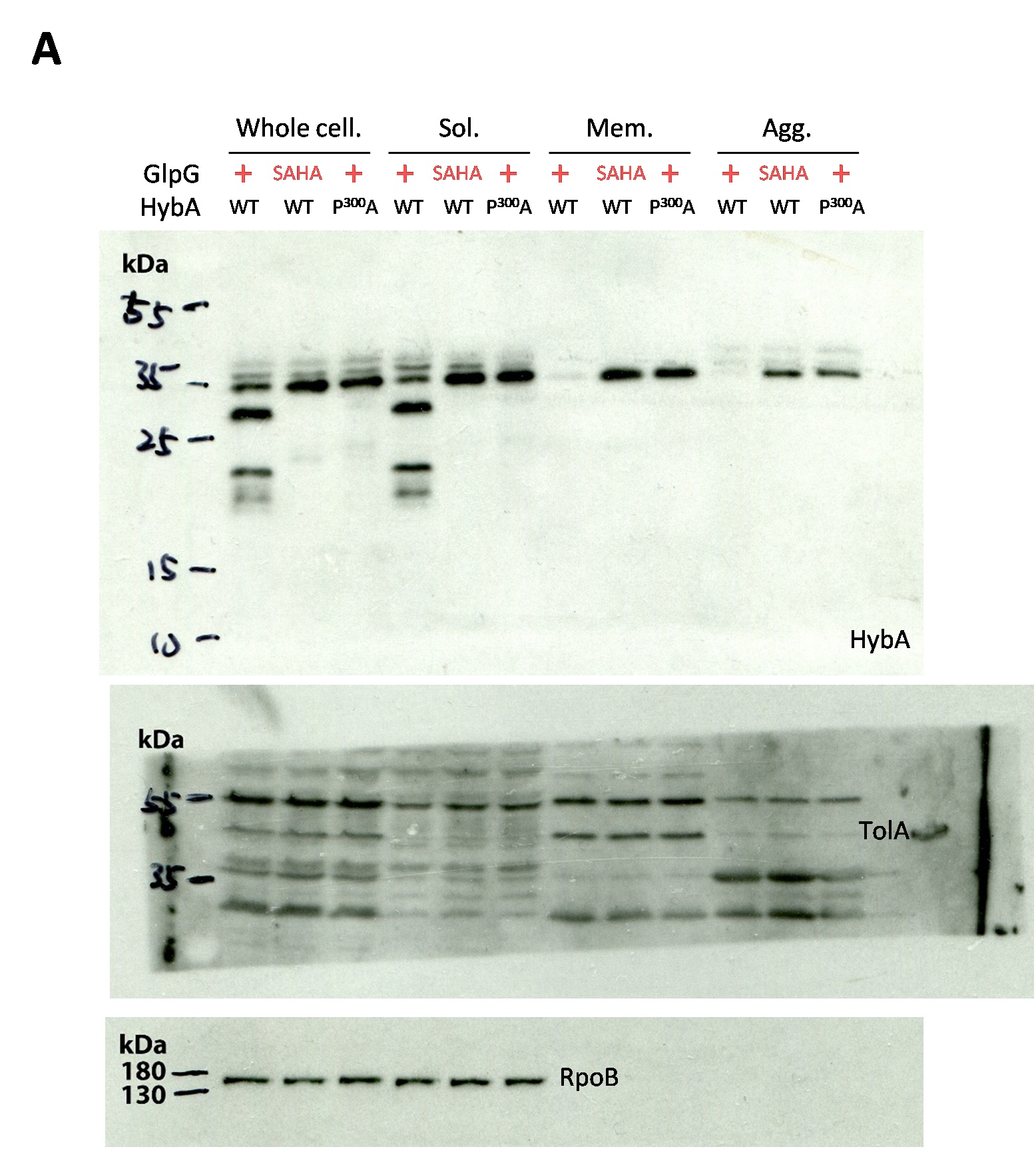

Supplement: Supplementary file 14 — Source Data for Figure 7 [file EMBJ-39-e102922-s013.jpg]
